# Engineering a suite of *E. coli* strains for enhanced expression of bacterial polysaccharides and glycoconjugate vaccines

**DOI:** 10.1186/s12934-022-01792-7

**Published:** 2022-04-21

**Authors:** Emily J. Kay, Marta Mauri, Sam J. Willcocks, Timothy A. Scott, Jon Cuccui, Brendan W. Wren

**Affiliations:** Department of Infection Biology, London School of Hygiene & Tropical Medicine, London, UK

**Keywords:** Biological conjugation, *Streptococcus pneumoniae*, Glycoengineering, Vaccine, PglB, Glycoconjugates

## Abstract

**Background:**

Glycoengineering, in the biotechnology workhorse bacterium, *Escherichia coli*, is a rapidly evolving field, particularly for the production of glycoconjugate vaccine candidates (bioconjugation). Efficient production of glycoconjugates requires the coordinated expression within the bacterial cell of three components: a carrier protein, a glycan antigen and a coupling enzyme, in a timely fashion. Thus, the choice of a suitable *E. coli* host cell is of paramount importance. Microbial chassis engineering has long been used to improve yields of chemicals and biopolymers, but its application to vaccine production is sparse.

**Results:**

In this study we have engineered a family of 11 *E. coli* strains by the removal and/or addition of components rationally selected for enhanced expression of *Streptococcus pneumoniae* capsular polysaccharides with the scope of increasing yield of pneumococcal conjugate vaccines. Importantly, all strains express a detoxified version of endotoxin, a concerning contaminant of therapeutics produced in bacterial cells. The genomic background of each strain was altered using CRISPR in an iterative fashion to generate strains without antibiotic markers or scar sequences.

**Conclusions:**

Amongst the 11 modified strains generated in this study, *E. coli* Falcon, Peregrine and Sparrowhawk all showed increased production of *S. pneumoniae* serotype 4 capsule. Eagle (a strain without enterobacterial common antigen, containing a GalNAc epimerase and PglB expressed from the chromosome) and Sparrowhawk (a strain without enterobacterial common antigen, O-antigen ligase and chain length determinant, containing a GalNAc epimerase and chain length regulators from *Streptococcus pneumoniae*) respectively produced an AcrA-SP4 conjugate with 4 × and 14 × more glycan than that produced in the base strain, W3110. Beyond their application to the production of pneumococcal vaccine candidates, the bank of 11 new strains will be an invaluable resource for the glycoengineering community.

**Supplementary Information:**

The online version contains supplementary material available at 10.1186/s12934-022-01792-7.

## Background

Bioconjugation is a burgeoning field due to its promise of lower cost vaccine production that is scalable and has fewer downstream purification steps than current chemical conjugation methods. Instead of separately purifying protein and glycan moieties and coupling them via chemical/enzymatic reactions requiring the development of multiple processes and consequently increasing quality control steps [1], a conjugate vaccine can be readily produced within a bacterial cell [2]. The field of bioconjugation was initiated by the characterisation of the *Campylobacter jejuni* PglB oligosaccharyltransferase (OST) [3, 4], which modifies a number of proteins in its native *C. jejuni* host via N-linked glycosylation [5–7], and its functional transfer to the periplasm of *E. coli* cells [8]. This singular proof of principle study heralded a new era in glycoengineering with the potential to clone and express glycosylated proteins (and other glycostructures such as capsular polysaccharides and lipooligosaccharides) in *E. coli* cells. Subsequently, advances have been made in identifying the consensus sequence necessary for modification of a protein by CjPglB [9] and incorporating this short amino acid sequence, referred to as a ‘Glyco tag’, into virtually any protein so that it would become a substrate for PglB-mediated glycosylation [10]. The coupling enzyme, PglB, has also been engineered for increased efficiency and broader substrate specificity, so that glycans with a galactose reducing end (the starting residue of a polymerized glycan) may be transferred in addition to the *N*-acetylated reducing end sugars preferred by the native *C. jejuni* PglB (*Cj*PglB) [11]. Many other orthologues of *Cj*PglB have been identified and analysed for improved efficiency and diverse substrate specificity [12, 13], in addition to different *N*-glycosylation site preference [14]. As yet no *Cj*PglB orthologues have been shown to transfer glycans with a glucose reducing end sugar, although other glycosylation pathways have been investigated that may be able to. For instance, *O*-glycosylation transfers lipid-linked glycans, in the periplasm, to serine or threonine residues within a protein. *O*-linked OSTs such as PglL [15–17] and PglS [18] have broad substrate specificity and can transfer glycans starting with a glucose residue but have more constrained acceptor requirements. There is also an alternative *N*-glycosylation system, discovered in *Actinobacillus pleuropneumoniae,* where NGT transfers nucleotide-activated monosaccharides of glucose to the acceptor protein, in the cytoplasm [19, 20]. Recent developments in protein glycosylation have been reviewed elsewhere [21, 22].

Previous work from our group and others has shown recombinant expression of *S. pneumoniae* polysaccharides in *E. coli* [23–26] and generation of glycoconjugate vaccine candidates [18, 27, 28]. Production of a glycoconjugate vaccine in vivo hinges on the ability to express a combination of glycan antigen, OST and acceptor protein under optimal conditions. Factors such as growth media, induction conditions and supplements (co-factor and nucleotide activated sugar substrates) have been varied to optimise glycoconjugate production [29].

There is also an increased emphasis on rationally altering the bacterial cell used for production. ‘Chassis engineering’ has been used extensively in the field of biopolymers to refine production [30]. For glycoconjugates this includes removal of interfering factors that may compete for common resources, such as WaaL, the O-antigen ligase [31], which competes with PglB for tranfer of the glycan substrate, and the enterobacterial common antigen (ECA [32]), which subtracts common building blocks for the synthesis of the glycan antigen and is built on the same lipid anchor. Improvement of metabolic flux by removal of metabolic components, altering NADPH/NADH ratio to improve glycolytic flux, varying flux through the glyoxylate cycle [33], phosphotransferase system [34], or blocking glycolysis and the pentose phosphate pathway [32], have all been shown to improve glycoconjugate yield. Precursor engineering, increasing the availability of initial sugar [35] also improved yields as did integration of exogenous components into the *E. coli* chromosome. Enhancement of glycoprotein production was seen upon integrating *CjpglB* [36], or the glycan synthesis locus [37], onto the chromosome. However, as yet there is no consensus or consistent bank of *E. coli* strains for use in glycoengineering.

There are many methods for making mutations in *E. coli* including scarless methods that do not rely on antibiotic selection, such as CRISPR [38, 39] and I-SceI endonuclease, an alternative way of creating double strand breaks [40]. Though I-SceI does not work well for all genes and modifications [41], and requires the introduction of I-SceI sites, resulting in additional steps compared to the CRISPR technology. Gene gorging is another method but relies on post recombination screening to identify mutants, which can be time consuming [42]. Furthermore, there is also the possibility of introducing an antibiotic cassette and then removing it later leaving a scar, for example with the *flp*/*frt* recombinase [43] or *Cre*/*lox* system [44].

The production of inexpensive, broad coverage vaccines against *S. pneumoniae* is a global imperative. There are currently several licensed vaccines for use against *S. pneumoniae*: one polysaccharide vaccine Pneumovax (PPSV23); and three pneumococcal conjugate vaccines (PCV): Prevnar 7 (PCV7), Prevnar 13 (PCV13) and Synflorix (PCV10) are widely used, with many more in clinical development [45]. Indeed, in the past year the USA has licensed two higher valent vaccines [46], Pfizer’s Prevnar-20 [47] and Merck’s Vaxneuvance (PCV-15) [48]. According to the CDC there are 100 identified serotypes as of 2020, and whilst conjugate vaccines reduce both carriage and invasive pneumococcal disease caused by the vaccine serotypes, polysaccharide-only vaccine PPSV23 has no effect on carriage [49]. The result of this is insufficient serotype coverage for many geographical locations. In Africa, since the introduction of PCV10/PCV13, vaccine efficacy against invasive pneumococcal disease has been reported to be 30–80% whilst the prevalence of targeted serotypes dropped by 35–90% [50]. In Africa and Asia, where morbidity and mortality rates of pneumococcal disease are globally the highest, serotypes included in existing PCV formulations still account for 49–88% of deaths [51]. This could in part be due to access and cost of the current vaccines [52].

In this study we use recombinant *S. pneumoniae* serotype 4 capsule expression and glycan transfer as a model system to develop a bank of rationally designed *E. coli* strains for glycoconjugate vaccine production, using CRISPR recombination. The newly developed strains have several features, including reduced endotoxin toxicity (deletion of *lpxM*), more faithful glycan production (deletion of *wecA*) and increased glycan availability for protein conjugation (removal of the O-antigen ligase WaaL). Chain length determinants for *S. pneumoniae* were also added to increase glycan polymer length and the OST (*CjpglB*) was integrated into the chromosome to reduce metabolic burden on the cell by reducing the number of plasmids and antibiotics used to selectively maintain them. Due to the broad applicability of these mutations, these strains will provide a valuable and practical resource for the glycoengineering community.

## Results

**Fig. 1:**
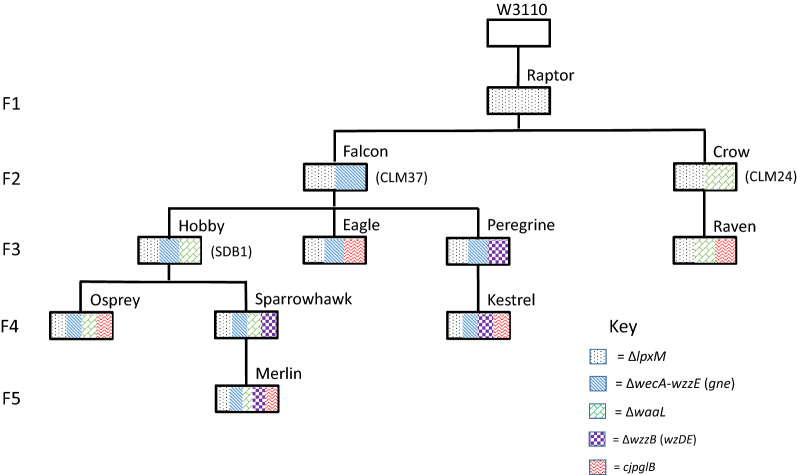
Engineering a family of glycoengineering strains. The strains are all produced from the progenitor *E. coli* strain W3110. Subsequent CRISPR mutations are made as designated in the key. Genes in parentheses denote integrations whilst Δ indicates deletions. Strain names are shown above the boxes, which are arranged in a family tree to denote the background strain where successive mutations were introduced, in a series of generations denoted on the left hand side (F1-F5). Strains equivalent to known glycoengineering strains CLM24, CLM37 and SDB1 are also indicated at the side of the boxes in parentheses

In order to provide a versatile suite of glycoengineering strains for various downstream application, CRISPR mutagenesis was used as a markerless tool for the introduction of sequential mutations of the base *E. coli* strain, W3110 (Fig. 1 and Additional file 1: Table S2), a widely used lab strain naturally lacking the native O-antigen. All strains were subject to PCR verification of all mutations (Additional file 1: Figure S1) and sequencing (Eurofins Genomics). Growth curves were compared before addition of accessory plasmids (Additional file 1: Figure S2).

### Modifying lipid A structure for reduced endotoxicity

**Fig. 2:**
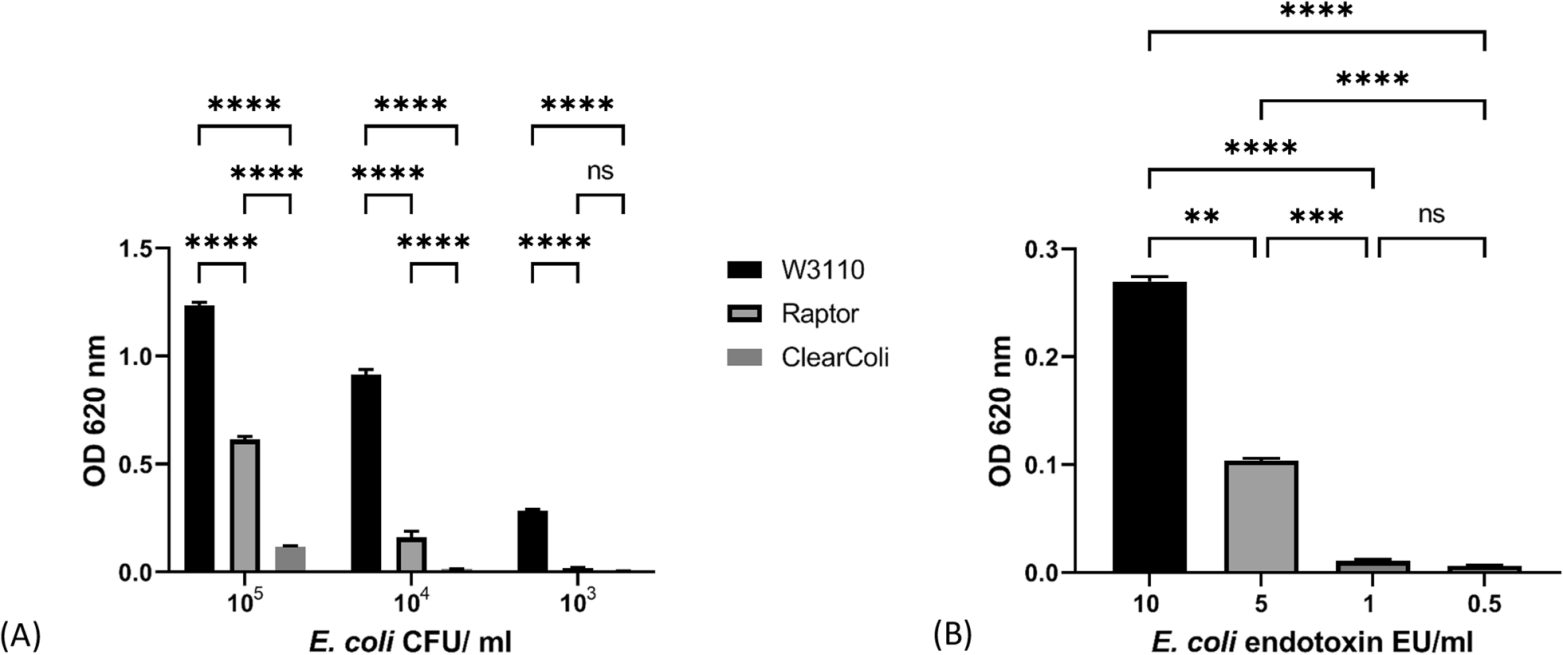
TLR4 activation assay. Colourimetric response of HEKBlue cells to TLR4 activation at different dilutions of W3110, Raptor and ClearColi cells (**a**) or *E. coli* 0111:B4 endotoxin dilutions measured in endotoxin units (EU) per ml (**b**). All dilutions were represented in triplicate and mean, blank-adjusted values plotted, with error bars representing standard error of the mean. For **A** significance was determined using two-way ANOVA with Tukey’s multiple comparison test. Only significance for pairwise comparisons between samples, at each dilution are displayed. A full list of all comparisons is shown in Additional file 1: Table S5. For **B** significance was determined using one-way ANOVA with Tukey’s multiple comparison test. For both a and b ns P > 0.05, P** < 0.01, P*** < 0.001, P**** < 0.0001

Traces of endotoxin in injectable or implantable products are a major concern in the biotech industry as their presence triggers an immune response, which may result in an anaphylactic shock in a few subjects, making it imperative to find cost-effective strategies to reduce its presence to assure product safety. Due to its universal appeal for reducing endotoxicity, LpxM was chosen as the progenitor mutation in the *E. coli* strain collection for glycoconjugate vaccine production (Fig. 1). LpxM is a Kdo2-lauroyl-lipid IVA myristoyl-ACP acyltransferase, the removal of which produces endotoxin that significantly reduces dendritic cell activation [53]. The strain was designated “Raptor” (Fig. 1) and shown to trigger significantly less activation of human Toll-like Receptor 4 (TLR4) in a HEK-Blue™ cell assay (Fig. 2a) than W3110 across a range of CFU/ml. Raptor displayed an intermediate phenotype between W3110 and ClearColi, which is a commercially available strain that contains a radically altered LPS recommended for its lack of endotoxic activity [54]. The colorimetric change is not significant between Raptor 10^4^ and ClearColi 10^5^ CFU/ml (Additional file 1: Table S5) which suggests a similar level of TLR4 activation. Whereas the difference between Raptor 10^4^ and W3110 10^3^ CFU/ml is still significant. Calculations for CFU/ml are shown in Additional file 1: Figure S3. TLR4 activation across a range of *E. coli* O111:B4 endotoxin dilutions are shown in Fig. 2b. W3110 10^3^ CFU/ml produce a similar level of activation to 10 endotoxin units (EU)/ml, whereas 10^4^ Raptor CFU/ml produce a level of activation comparable to 6 EU/ml. HEK-Blue cells are a human reporter cell line used here as a proxy for innate immune response to endotoxin.

**Fig. 3:**
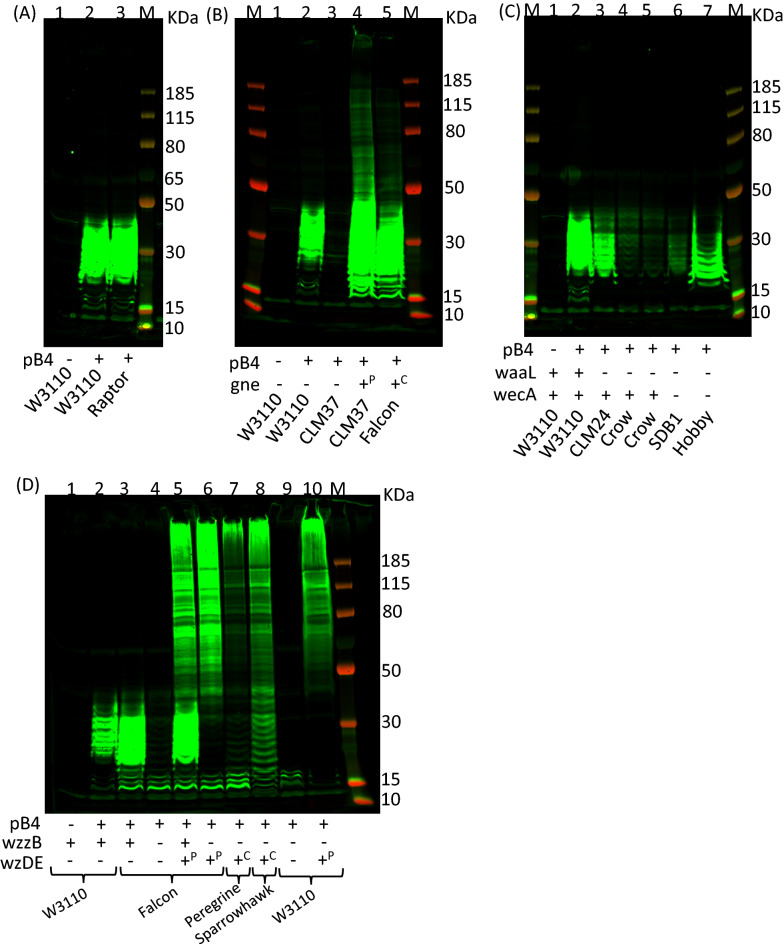
Immunoblot of recombinantly expressed *S. pneumoniae* capsule in *E. coli*. Lysed, whole cell samples were separated by SDS–PAGE on a Bolt 4–12% bis–tris gel run with MOPS buffer. Glycan was detected using anti-serotype 4 primary antibody and anti-rabbit fluorescent secondary antibody. *M* molecular weight marker PageRuler Plus. Presence or absence of pB4 recombinant *S. pneumoniae* serotype 4 capsule is denoted below the lanes as ± respectively, as are the strain names. **a** *lpxM* deletion. **b** wecA deletion and insertion of g*ne*. Presence or absence of *gne* is denoted below the lanes as ± respectively;  + ^P^ denotes *gne* is provided on pMAF12 plasmid,  + ^C^ denotes chromosomal integration of *gne* in place of *wzzE-wecA*. **c** *waaL* deletion. The presence or absence of *waaL* and *wecA* are denoted below the lanes as ± respectively. In the case of Hobby strain *gne* is also integrated on the chromosome. **d** Chain length modification. The presence or absence of *wzzB* and *wzDE* are denoted below the lanes as ± respectively. In the case of WzDE + ^P^ denotes the presence of pEXT21:*wzDE* and + ^C^ indicated that *wzDE* are integrated in place of *wzzB* on the chromosome

To show that there was no obvious impact of removing *lpxM* on recombinant polysaccharide expression, *S. pneumoniae* serotype 4 capsule was expressed within this strain by introducing plasmid pB4 (Fig. 3a). There was no discernible difference in banding pattern or strength of signal to OD_600 nm_-matched samples of Raptor pB4 (lane 3) when compared to W3110 carrying pB4 (lane 2).

#### Enhancing availability of initial sugar of repeat unit

Previously, we have shown that deletion of the initiating sugar transferase, *wciI* does not impact the expression of recombinant SP4 capsule, unless *wecA* is also removed from the host strain [25]. This suggests that in the background strain W3110 most of the recombinant glycan produced starts with GlcNAc instead of the SP4 native GalNAc. This is the result of the native *E. coli* WecA, transferring GlcNAc onto the lipid carrier undecaprenol pyrophosphate [55, 56]. Therefore, we tested whether removal of *wecA* coupled with addition of the *gne* [57], UDP N-acetylglucose epimerase from *C. jejuni*, could enhance SP4 production. Gne converts UDP-GlcNac to UDP-GalNAc thereby increasing the available pool of the initial sugar required for SP4 capsule synthesis. The strain “Falcon” was produced where the genes *wecA-wzzE* in the synthesis of ECA synthesis locus were replaced with *gne*, inserted under control of the native, constitutive *wecA* promoter, in the Raptor background. *wzzE* encodes the enterobacterial common antigen chain length regulator which was also removed to prevent any interference with recombinant polysaccharide production. Figure 3b demonstrates that using a Δ*wecA* mutant strain CLM37 there is little production of serotype 4 polysaccharide (lane 3), until copies of the Gne epimerase are introduced by plasmid pMAF12 (lane 4). The chromosomal copy of *gne* in Falcon strain (lane 5) is sufficient to produce similar levels of recombinant polysaccharide to CLM37 pMAF12 pB4. Both these strains produce a higher banding pattern and brighter fluorescence than the original W3110 strain expressing SP4 (lane 2).

#### Removal of the O-antigen ligase, WaaL

W3110 produces no O-antigen due to the inactivated *wbbL* gene in the O-antigen cluster which prevents it from producing the native O:16 antigen [58]. The *E. coli* O-antigen ligase, WaaL, has relaxed specificity for its substrates and is able to transfer recombinant glycans to the Lipid A core *in lieu* of the native O-antigen, sequestering it from PglB, thus hampering protein coupling. As this unavailability of glycan would have a negative effect on glycoconjugate yield, *waaL* was removed in the Raptor and Falcon strains to create the “Crow” and “Hobby” strains, respectively (Fig. 1). To our surprise the Crow strain (Fig. 3c lane 4 and 5) produces much less SP4 glycan than CLM24 (lane 3), which is also a *waaL* knockout strain. SDB1 (lane 6) has both *wecA* and *waaL* mutations but produces less glycan than its Hobby strain counterpart (lane 7), which has both mutations in addition to the *gne* insertion coding for an epimerase and *lpxM* depletion.

**Fig. 4:**
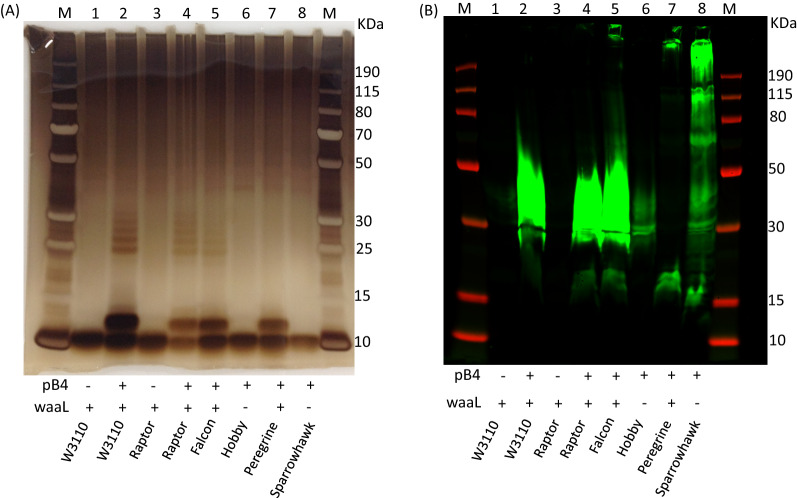
SDS-PAGE of lipid extracts from *E. coli* strains recombinantly expressing *S. pneumoniae* capsule. Lipid extracts were separated by SDS–PAGE on a Bolt™ 4–12% bis–tris gel run with MES buffer. **A** Silver stain; **B** immunoblot where glycan was detected using anti-serotype 4 primary antibody and anti-rabbit fluorescent secondary antibody. *M* molecular weight marker PageRuler Plus. Presence or absence of pB4 recombinant *S. pneumoniae* serotype 4 capsule and *waaL* are denoted below the lanes as ± respectively, as are the strain names

To demonstrate that deletion of the O-antigen ligase prevents attachment of the recombinant SP4 polysaccharide to the lipid A core, a lipid extraction was conducted (Fig. 4). As can be seen from Fig. 4a a shift in the Lipid A core followed by banding pattern in the silver stain is apparent only for strains W3110, Raptor, Falcon and Peregrine that contain pB4 (lanes 2, 4, 5 and 7), whereas Hobby and Sparrowhawk containing pB4 do not show this shift (lanes 6 and 8) despite polysaccharide being produced, as evidenced from the same samples probed via western blot with SP4 specific antisera (Fig. 4b). Negative controls of the W3110 and Raptor strains without pB4 (lane 1 and 3) show that this pattern is not observed in the absence of pB4. Additionally, removal of LpxM does not appear to alter how the lipid runs on an SDS-PAGE gel or by silver staining. The Peregrine strain does not have the same banding pattern as strains Raptor and Falcon, given the chain length of the polymer has been altered in this strain as can be seen by the western blot where the majority of the polymer appears to be stuck in the well or runs very close to the top of the gel, whereas on the silver stain there is high background and this cannot be seen.

#### Chain length regulation

As can be seen from Fig. 3a–c, there is a limited range of bands present on the western blots, below 50 kDa. This limited range is partially overcome by addition of the Gne epimerase (Fig. 3b lane 4 and 5), however the bands below 50 kDa are still much brighter than those above. The pB4 construct does not contain chain length regulators from the *S. pneumoniae* capsule locus. To explore the role of chain length regulation in pB4 the *wzD* and *wzE* genes from TIGR4 (a wild type serotype 4 *S. pneumoniae* strain) were initially cloned into a low copy number, IPTG-inducible vector, pEXT21 [59], and then inserted onto the chromosome in place of the native *E. coli* chain length regulator *wzzB* under a rhamnose inducible promoter [60] using CRISPR. The addition of rhamnose was not necessary to induce expression (data not shown). When cloning the *wzDE* genes into the pTarget plasmid it was not possible to find a clone without a frame shift mutation in *wzE*. Due to this, the frame shift was repaired by a second round of CRISPR cloning where a corrected portion of *wzE* was grafted on top of the original mutation to restore the *wzE* gene, whilst removing the CRISPR cut-site by replacement with a synonymous codon sequence (Additional file 1: Figure S4).

Removal of the *E. coli* O-antigen chain length determinant (CLD), *wzzB*, from Falcon or W3110 has a negative impact on recombinant capsule expression, where only faint banding can be seen near the bottom of the gel (Fig. 3D, lane 4 and 9). This can be restored by expressing pEXT21-encoded *wzDE* (lane 6 and 10). When plasmid pEXT21:wzDE is introduced into Falcon strain without removing *wzzB*, then the limited repeats can be seen in addition to the higher polymer repeats (lane 5). This is not seen in the absence of *wzzB*. When pEXT21:*wzDE* is introduced into W3110 containing pB4 plasmid the strain grows slowly and lyses before reaching stationary phase, showing toxicity in the presence of *wzzB* (data not shown). Strain Peregrine (lane 7) is produced from Falcon strain with *wzDE* added to the chromosome in place of *wzzB* and Sparrowhawk strain (lane 8) is *wzDE* added to Hobby strain. Peregrine strain has a functioning WaaL and Sparrowhawk does not (Fig. 1).

### PglB-mediated conjugation of glycan to protein

**Fig. 5:**
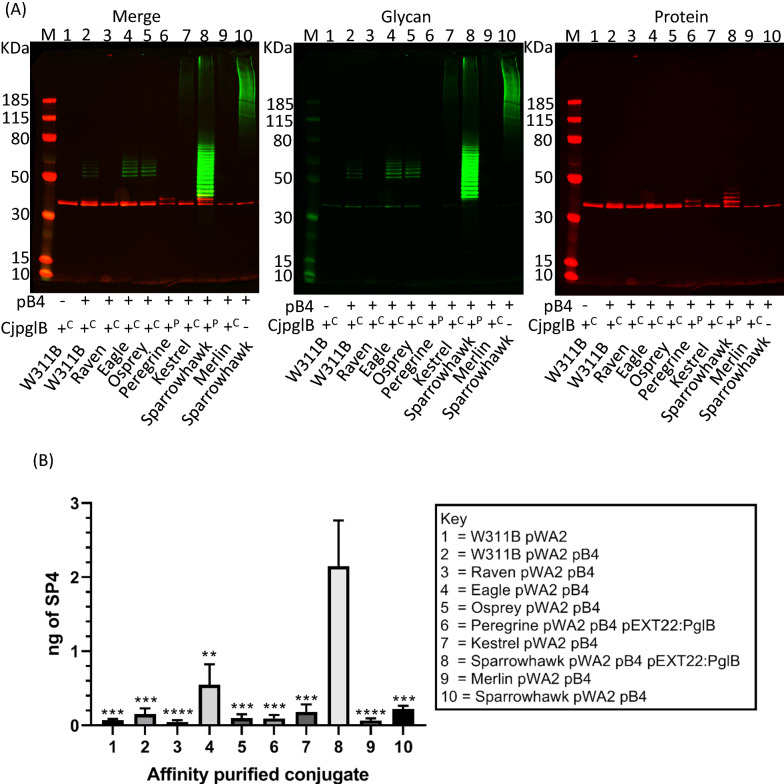
Analysis of purified AcrA-SP4 Glycoconjugates produced from different *E. coli* strains. **A** 5 µg protein separated by SDS-PAGE 4–12% bis–tris gel run with MOPS buffer. Glycan was detected using anti-serotype 4 primary antibody and anti-rabbit 800 fluorescent secondary antibody (green). Protein was detected using anti-His primary antibody and anti-mouse 680 fluorescent secondary antibody (red). *M* molecular weight marker PageRuler Plus. All strains contained plasmid pWA2 which expressed *C. jejuni* AcrA protein at 40 kDa. Presence or absence of pB4 recombinant *S. pneumoniae* serotype 4 capsule and *cjpglB* are denoted below the lanes as ± respectively, as are the strain names. In the case of *cjpglB* + ^P^ indicates the presence of plasmid pEXT22:pglB and + ^C^ indicates cjpglB integrated into the chromosome. For strain W311B *cjpglB* is under control of P_tac_ promoter, rather than the Anderson 0.1 promoter. **B** Quantification of capsular polysaccharide production by sandwich ELISA. Biological triplicate samples were processed in triplicate. Values for amount of capsule produced were interpolated from a standard curve of purified type-specific capsular polysaccharide (Statens Serum Institut, Denmark) and expressed as capsule produced per 2.5 µg of protein. Data are presented as the mean of biological replicates with error bars denoting the standard error of the mean. Significance was determined using a one-way ANOVA with Tukey’s multiple correction. Asterisks above sample bars denote significance relative to bar 8 (Sparrowhawk pWA2 pB4 pEXT22:PglB) **P < 0.005, ***P < 0.0005, ****P < 0.0001

PglB is a multi-transmembrane OST enzyme that shows toxicity when expressed in *E. coli* and as such often has a negative impact on cell growth rate once induced [61]. C*jpglB* was inserted, using CRISPR, into the chromosome between *gidB* and *atpI* which was shown to be a stable integration site in previous studies [62]. Transcription of *CjpglB* was placed under the control of an Anderson promoter of strength 0.1 [63]. Five integrated *CjpglB* strains were created from strains Crow, Falcon, Hobby, Peregrine and Sparrowhawk to produce Raven, Eagle, Osprey, Kestrel and Merlin strains respectively (Fig. 1). *C. jejuni* AcrA was chosen as an acceptor protein to evaluate functionality of these strains as it is a natural PglB substrate. Initially *C. jejuni* heptasaccharide, encoded on plasmid pACYC*pgl*, was transferred to inspect site occupancy of the two glycosylation sites of AcrA. Additional file 1: Figure S5 shows that all the strains transfer *C. jejuni* heptasaccharide to AcrA, but not as efficiently as the W311B strain, which contains a chromosomally-encoded *CjpglB* under the control of the *lac* operator. Next *S. pneumoniae* serotype 4 was transferred (Fig. 5) to AcrA. As the Anderson promoter driven chromosomal *CjpglB*s were not strongly transcribed, pEXT22-PglB was used as a comparison in strains Peregrine and Sparrowhawk (lane 6 and 8). These plasmid-based, IPTG inducible *CjpglB*s promoted higher glycosylation of AcrA as shown by the band shifts in the anti-his antibody channel (red). Peregrine (lane 6) performed better than Kestrel (lane 7), and Sparrowhawk (lane 8) better than Merlin (lane 9) where the difference was *CjpglB* on plasmid vs chromosome in each case. Raven (lane 3) and Merlin (lane 9) strains showed no transfer, but for the other strains there are band shifts visible in the red, anti-his antibody channel, above where the unglycosylated, AcrA would run, as seen in the AcrA only lane (lane 1). A further protein sample was purified from strain Sparrowhawk, containing AcrA and pB4 without *CjpglB*. No visible red band is present above the unglycosylated, AcrA (lane 10), but there is some co-purified glycan that is not attached to the protein by PglB. This glycosylation pattern looks different to Sparrowhawk with PglB (lane 8) where a ladder of banding in the anti-his channel is also seen.

To quantify the glycan content of each glycoconjugate, a sandwich ELISA was performed whereby his antisera was used to coat an ELISA plate as a capture antibody before 2.5 µg of protein conjugate was bound and probed with SP4 antisera. A standard curve of purified polysaccharide from *S. pneumoniae* was used as a reference for glycan content. Figure 5b shows that the Eagle strain outperformed the original W311B strain with 4 × more glycan attached, but out of all the Sparrowhawk strain showed the clearest improvement with approximately 14 × as much glycan attached.

## Discussion

In this study rational design employing CRISPR in combination with λ red was used to produce sequential scarless mutations in the background W3110 *E. coli* strain, without the need of introducing antibiotic resistance markers. Removing antibiotic resistance markers allows for greater flexibility when adding in extra plasmid-borne components for glycoconjugate production, as well as being a necessary requirement for vaccine producing strains. Without the need for a selection marker, it is also possible to knockout and insert genes at the same time, recombining them at precise locations. This includes modifying promoters to fine tune transcription and the relative expression of glycans or proteins. Overall, CRISPR is a simple, flexible tool that has been instrumental in creating this bank of mutant strains.

It is widely recognised that the lipid A portion of LPS is responsible for its proinflammatory properties. The lipid A moiety of LPS activates Toll-like receptor 4/myeloid differentiation factor 2 complex (TLR4/MD2) resulting in cytokine production, which can lead to endotoxic shock [64]. Proteins and conjugates purified from *E. coli* are contaminated with LPS, thus removing leftover traces of endotoxin is necessary for vaccine safety and for passing regulatory quality controls despite being an expensive and time consuming process [65]. An *lpxM* deletion creates a relatively minor change in the kdo core of lipid A, by transfer of myristate from myristoyl-acyl carrier protein (ACP) to Kdo_2_-(lauroyl)-lipid IV(A) [66, 67]. Previous studies have shown a marked reduction in TNF-α production when *lpxM* is removed, but normal bacterial growth [53]. In this study, HEK-Blue cells were used as a reporter system to detect the presence of functional endotoxin. HEK-Blue cells stably express TLR4 along with other genes from the TLR4 pathway. Additionally, they co-express an NF-κB-inducible secreted embryonic alkaline phosphatase (SEAP) reporter gene, which allows for a colorimetric detection of TLR4 activation. This study showed a significant reduction in TLR4 activation by the Raptor strain, where *lpxM* has been removed using CRISPR mutagenesis, compared to the background strain W3110. Other groups have made several more mutations in *lpxL*, *lpxM*, *pagP*, *lpxP* and *eptA* [54]. However, these mutations were found to result in growth defects under certain conditions. There are also commercially available strains such as BL21 DE3 ClearColi (Lucigen) strain with > 97% reduction in endotoxic activity. This study has shown the Raptor strain has an intermediate phenotype between W3110 and ClearColi cells for TLR4 activation. Using the CRISPR method it would be possible to incrementally and rationally create additional mutations to decrease endotoxicity further. However, a balance would have to be struck between overall yield, efficiency of glycoconjugate production, and level of endotoxin produced by the cell. It is hoped that conjugate vaccines produced from strains without *lpxM* will have far less endotoxic activity after purification.

Once endotoxicity was reduced, the Raptor strain was selected as a base strain to make a series of knockout and insertion mutations designed to firstly, increase the amount of recombinant SP4 glycan produced and secondly, to increase the availability of the glycan for transfer to protein using the OST enzyme PglB. The lipid extraction, Fig. 4A, shows that in the *lpxM* mutant, SP4 is still attached to lipid A, and western Fig. 3a shows that similar amounts of SP4 are produced in this strain compared to W3110.

A major consideration in producing recombinant glycans is the availability of their monosaccharide components in the host strain. It has previously been demonstrated that GalNAc is limited in *E. coli*, so the addition of GlcNAc-GalNAc epimerase Gne improves recombinant SP4 polysaccharide production as can be seen in Fig. 3b. Removal of *wecA* serves a dual purpose, both to ensure the reducing end sugar is not replaced with GlcNAc but also to remove production of ECA, which competes for limited supplies of und-P, the lipid carrier, upon which recombinant polysaccharides of Wzx/Wzy-dependent pathway are built. Antisera specific for SP4 recognises the repeat unit regardless of the initial sugar ensuring recognition of SP4 in both WecA positive and negative strains. WecA positive strains have been used to produce conjugate vaccines, previously shown to be protective [27, 28], suggesting that the reducing end sugar may not be instrumental for triggering an immune response. Und-P needs to be recycled as supply is limited and multiple glycan pathways rely on it including peptidoglycan biosynthesis [68, 69]. In strains where the recombinant glycan cannot be removed from und-P and added to either lipid A where WaaL is still present, or to a carrier protein where PglB is added, there may be a limit to the amount of glycan produced. This appears to be the case for the Crow and Hobby strains. The Hobby strain produces more recombinant SP4 glycan than Crow, possibly due to the removal of WecA in the Hobby strain, which increases the availability of und-P as ECA is no longer produced, combined with the extra availability of GalNAc by addition of the Gne epimerase.

There are limited inducible promoter systems available that do not require the insertion of more accessory genes. This reduces the ability to fine tune the expression of individual system components when more than one relies upon the same induction strategy. Constitutive expression of non-toxic components can be beneficial. To this end, *wecA* was replaced with *C. jejuni gne* under control of the native *wecA* promoter, by inserting *gne* at the starting methionine codon of *wecA*. Replacing *wecA* with *gne* had a positive impact on the amount of recombinant polysaccharide produced.

WaaL, the O-antigen ligase, also competes with PglB for the glycan substrate that could be attached to a protein for glycoconjugate vaccine production. There are some instances where displaying the recombinant glycan on the surface may be desirable, for example, OMVs or killed bacterial vaccine [26, 70]. From the library assembled in this study, WaaL positive or negative strains can be selected dependent on downstream application.

*S. pneumoniae* serotype 4 produces capsular polysaccharide in a Wzx/Wzy dependent manor. In Wzx/Wzy-dependent polysaccharide biosynthesis, a single repeat unit is transported across the cytoplasmic membrane into the periplasm by the flippase (Wzx), the polymerase (Wzy) then extends the repeats via the polymerizing linkage, and the range of repeat units is regulated by the chain length regulator (Wzz) [71]. For plasmid pB4, the native chain length regulators from *S. pneumoniae* were not added during initial cloning [25]. In S*. pneumoniae* there are four regulators of capsule production in a phosphokinase relay that are highly conserved among all serotypes that produce Wzx/Wzy dependent capsule. Wzh and Wze are required for capsule production and virulence, whereas a Wzg deletion mutant was able to produce a partial capsule [72]. Only the last two, WzDE, are required for chain length modification [73]. It is apparent that chain length regulators can compensate between different glycans and bacterial species [74]. In this study it appears that the chain length regulator of the *E. coli* O-antigen in some way interacts with the flippase and polymerase of *S. pneumoniae* to modulate the number of capsule repeat units in the polymer. As can be seen from Fig. 3D without WzzB or *S. pneumoniae* chain length regulators, very little visible glycan is produced indicating very few repeat units (lane 4 and 9). When WzzB is present in conjunction with WzDE there seems to be competition between the two systems. In the Falcon strain (lane 5) this can be seen as two distinct patterns of polymer on the western blot: one with the reduced polymer banding of WzzB and one with the higher banding pattern of WzDE. For W3110 the strain containing WzDE and WzzB grew very slowly so there was clearly a toxic effect, which was ameliorated by removal of WzzB. This was more pronounced in W3110 than in Falcon, which may be due to WecA providing N-acetylglucosamine as the reducing end sugar which would be a native substrate for the WzzB [75]. Further investigation would be needed to untangle this issue.

In chemically conjugated vaccines a polysaccharide to protein ratio is selected for all serotypes to be consistent regardless of known differences in immunogenicity for the different serotypes [76]. There is some evidence that the length of polymer affects immunogenicity for some strains [77, 78]. However, synthetically generated short repeats were found to generate a comparable immune response when compared to Prevnar (PCV13) with the exception of serotype 14 [79]. Increasing polymer length is a way to increase the glycan to protein ratio of the conjugate and also maintain secondary structure epitopes, where present, which may boost immunogenicity [80]. It is unknown what the optimum number of capsule repeat units for generating a strong and lasting immune response would be for each serotype. It is likely, however, that the optimum length will be serotype specific and also dependent on the protein to which the glycan is conjugated. These new strains provide flexibility in the number of glycan repeat units available for conjugation so that this question may be addressed on a serotype specific basis. This has been exemplified recently in one of our former studies where the Falcon strain was identified for the optimal production of a SP4-ExoA conjugate [81].

Adding *CjpglB* to the chromosome under constitutive expression led to an increase in glycosylation of approximately 4 × in the case of the Eagle strain. The use of Anderson promoters [63] to constitutively express *CjpglB* removes reliance on induction systems for protein expression. However, the best performing strain overall was Sparrowhawk with *CjpglB* on a low copy number vector under IPTG expression control. This may be due to promoter strength and amount of PglB in the system. It will be possible eventually to alter the PglB promoter strength by subsequent rounds of CRISPR. This was initially not possible as the pTarget vector for delivery of gRNA and recombination template is high copy and, as such, a strong constitutive promoter was not tolerated for *CjpglB* expression. However, by reducing the plasmid burden on the cell it was possible to add a plasmidic copy of *CjpglB* into the Sparrowhawk strain, which led to a marked improvement of glycan to protein attachment under the conditions tested.

## Conclusions

This study shows the ease of using CRISPR technology to generate sequential mutations where genes can be removed, replaced, or modified at will without the use of antibiotic or other selective markers nor the generation of scar sequences. The strains presented in this work represent a base for further tailoring glycan expression and glycoconjugate vaccine production using bioconjugation. The eleven strains introduced here represent an improvement on existing *E. coli* base strains such as CLM24, CLM37 and SDB1. Moreover, they constitute an invaluable resource for the glycoengineering community, which could expand their application to a broader range of glycans and glycoproteins of bio technological interest.

## Materials and methods

### Strains and plasmids

A table of strains and plasmids used in this study can be found in the Additional file 1: Tables S1 and S2. *Escherichia coli* strains were cultured on LB agar or grown in LB broth or 2YP [soy peptone 1.6% (w/v), yeast extract 1.2% (w/v), NaCl 0.5% (w/v)] at 37 °C or 28 °C, with shaking. Antibiotics were added as necessary for plasmid maintenance: tetracycline 20 µg ml^−1^; ampicillin 100 µg ml^−1^; Streptomycin 80 µg ml^−1^; Kanamycin 50 µg ml^−1^.

### CRISPR mutation

The method and plasmids described by Jiang et al. were used for CRISPR mutation [38]. The spacer portion of the guide RNA (gRNA) was designed using Atum CRISPR gRNA design tool [82], to give 20 bp next to the protospacer adjacent motif (NGG) within the target region and added to the cRNA scaffold portion of the gRNA (see Additional file 1: Table S3). The gRNA was cloned into pTarget (Addgene 62226) downstream of the constitutive promoter. A recombination template was also cloned downstream of the gRNA consisting of at least 600 bp of flanking DNA either side of the excision/insertion site, along with any cargo sequence as appropriate. Recombination templates were either synthesised by IDT as gBlocks (Integrated DNA Technologies, Iowa USA) or stitched together using SOE PCR. For each recombination, two versions of pTarget were produced, one containing only the gRNA and one containing both the gRNA and recombination template.

Target strains were prepared by electroporation with pCas plasmid (Addgene 62225). The λ Red recombination system on plasmid pCas was induced using 0.2% arabinose, before cells were made competent using the Chung method [83]. Briefly, 5 ml of LB were inoculated with 50 µl of overnight cell culture, grown in LB with 0.2% arabinose at 28 °C. Cells were grown aerobically at 28 °C with shaking at 200 rpm until an OD_600_ of 0.4 was reached, before chilling on ice for 10 min. Cells were then harvested by centrifugation at 3000×*g* for 10 min and resuspended in 500 µl ice-cold TSB/DMSO medium [Tryptone 1% (w/v); Yeast extract 0.5% (w/v); NaCl 1% (w/v), MgSO_4_ 10 mM, MgCl_2_ 10 mM, DMSO 5% (v/v); Polyethylene glycol (MW 6000) 10% (w/v)]. Fifty µl of competent cells were incubated on ice with 100 ng of pTarget with and without the recombination template in two separate transformations. After 30 min of incubation SOC recovery media was added and cells were incubated for 1 h at 28 °C with shaking, before dilutions were plated on LB kanamycin, spectomycin and 0.1% glucose, and incubated overnight at 28 °C.

PCR was used to screen potential mutants (Additional file 1: Table S4) with FastTaq (Bioline) according to manufacturer’s instructions. Plasmid curing to cure pTarget plasmid, colonies were grown in LB plus Kanamycin and 0.5 mM IPTG overnight at 28 °C, then serially diluted and plated on Kanamycin plates. Patch plating, separately onto Kanamycin and Spectinomycin plates, was used to identify colonies that had lost pTarget but retained pCas. At this stage the strains were either induced with 0.2% arabinose and made competent again for further rounds of mutation, or the strain was cured of pCas. To cure pCas, colonies were grown at 37 °C overnight with no antibiotic selection, before serially diluting and plating on LB with no antibiotic at 37 °C. Patch plating on LB only at 37 °C and LB kanamycin at 28 °C was used to confirm that the plasmid had been lost.

### Expression of capsule and lysis of cells

*Escherichia coli* cultures grown for 16 h were diluted into fresh 2YP media to an OD_600_ of 0.03. For protein expression and conjugation, after 4 h of growth the media was supplemented with 0.5 mM IPTG and incubated at 28 °C for 24 h. Cultures were pelleted by centrifugation at 4000×*g* for 10 min 4 °C. At this stage pellets were either lysed for immunoblot or lipid extraction and silver stained as described previously [25, 84].

Pellets from strains expressing polysaccharide only were resuspended in PBS to OD_600 nm_ of 10 with 1 mg ml-1 lysozyme and benzonase 40 U ml^−1^ and boiled for 10 min before SDS-PAGE analysis. Conjugate samples were resuspended in 1.5 ml lysis buffer (50 mM NaH_2_PO_4_, 0.3 M NaCl and 10 mM imidazole, pH 7.5) with lysozyme and benzonase as above, and lysed using 5 × 40 s bead-beating rounds at 6.5 m/sec using a FastPrep-24 homogeniser (MP Biomedicals, Belgium), before His purification.

### His purification of protein

One ml Ni–NTA agarose (QIAGEN) was added to the supernatant. The slurry-lysate was incubated for 1 h at 4 °C with shaking then washed and eluted according to manufacturer’s instructions (QIA expressionist, QIAGEN) in buffer containing 250 mM imidazole.

Eluate was treated with Triton X-114 to reduce contamination with lipid-linked glycan [85]. Protein was quantified by Qubit fluorometric assay.

### SDS-PAGE and immunoblot

Lysed samples were mixed with SDS–PAGE sample buffer and separated on Bolt 4–12% bis–tris gels in MOPS or MES buffer (Invitrogen). Samples were electroblotted onto nitrocellulose membrane using an iblot 2 dry transfer unit (Invitrogen). Membranes were blocked for 1 h in PBS containing 2% w/v skimmed milk powder. *S. pneumoniae* Serotype 4 (SP4) rabbit anti-capsule antibody (Statens Serum Institut, Denmark) was used at a dilution of 1:1000 in PBS containing 2% w/v skimmed milk powder and 0.1% v/v Tween 20. After 1 h incubation with SP4 antibody, membranes were washed three times with PBS (0.1% Tween 20) and then incubated for 45 min with a secondary goat anti-rabbit IgG IRDye800 conjugate antibody at a dilution of 1: 10 000. Membranes were washed a further three times in PBS (0.1% Tween 20) and once with PBS before signal detection with the Odyssey LI-COR detection system (LI-COR Biosciences UK Ltd.).

### Sandwich ELISA

Wells of a MaxiSorp microtiter plate (Nunc, UK) were coated with 200 ng of Mouse anti-his IgG (Invitrogen) overnight at 4 °C. Wells were blocked with PBS 5% milk for 1.5 h before washing with PBS 0.05% Tween 20. Wells were then saturated overnight at 4 °C with 2.5 µg of glycoconjugate or protein only control. PBS was used as a negative control and a standard curve was generated using dilutions of purified pneumococcal polysaccharide (Statens Serum Institut, Denmark). After washing with PBS 0.05% Tween, wells were blocked with PBS 5% milk for 1.5 h, washed again and incubated with anti-capsule antisera (Statens Serum Institut, Denmark) at a dilution of 1: 1000 in PBS 2% milk for 1 h. After washing with PBS 0.05% Tween, goat anti-rabbit IgG HRP (abcam, UK) was added at a dilution of 1: 20,000 in PBS 2% milk for 45 min. After washing, TMB (ebioscience, UK) was added, the reaction was stopped with 2 M H_2_SO_4_. Indirect ELISA detection was performed using a SpectrMax iD5 microplate reader (Molecular Devices, UK) at an absorbance of 450 nm.

Data analysis and graphing were performed using GraphPad Prism version 9 for Windows. Three biological replicates were performed, with each sample probed in triplicate. A standard curve generated using purified type 4 polysaccharide (SSI, Denmark) and samples interpolated using sigmoidal 4PL where “x” is concentration.

### TLR4 activation assay

To quantify TLR4 activation, the HEK-Blue hTLR4 SEAP reporter assay (InvivoGen) was used. The cell line was cultured to ~ 80% confluency at 37 °C with 5% CO_2_ and maintained in DMEM Glutamax media (Thermofisher), supplemented with 5% fetal bovine serum; 100 µg ml^−1^ normocin (InvivoGen) and 1X HEK-Blue selection solution (InvivoGen). Cells were washed and detached by gentle resuspension with sterile PBS and enumerated with a haemocytometer. A solution of 2.5 × 10^4^ cells per 180 µl HEK-Blue detection media (InvivoGen) was aliquoted into each well of a flat 96-well microplate containing 20 µl/well of each bacterial strain, purified *E. coli* 0111:B4 endotoxin (ThermoFisher Scientific) or untreated control as described in HEK-Blue detection media. The plate was incubated at 37 °C overnight before the supernatant was transferred to a fresh 96-well microplate and absorbance recorded at 620 nm using a SpectraMax M3 (Molecular Devices) spectrophotometer.

## Supplementary Information

**Additional file 1: Table S1.** Plasmids used in this study. **Table S2.** Strains used in this study. **Table S3.** N_2_0 specific sequence of gRNA and protospacer adjacent motif (PAM). **Table S4.** Primers for mutant confirmation. **Table S5.** 2 way ANOVA multiple comparisons for TLR4 activation assay. **Figure S1.** PCR Verification of mutations within strains. Overnight cultures of each strain were lysed using Chelex 100 (BioRad). PCR was performed with Platinum Green Hot start PCR mastermix (Invitrogen), in 20 µl reaction, according to manufacturer’s instructions, using Tm determined by NEB Tm calculator. Ten µl was run on an agarose gel alongside Hyperladder 1 Kb marker (Bioline) and visualized with gelRed nucleic acid stain (biotium). *M* marker; *B* negative control no template PCR. **Figure S2.** Growth curve of all strains. Overnight cultures of all strains were used to inoculate fresh LB broth to an OD_600_ of 0.03. Cultures were incubated at 37 °C with shaking and OD_600_ measurements taken at regular intervals with a final reading taken at 24 h. Strains did not contain plasmids. Strains depicted in red contain *pglB *and grow slightly slower than those without and reach a slightly lower final OD_600_. W3110 is included as a wild type comparison. The experiment was performed in triplicate and the data shown are mean values with error bars depicting standard error of the mean. **Figure S3.** Colony forming units for TLR4 activation assay. Overnight cultures of W3110, Raptor and ClearColi strains were matched for OD_600_ value, serially diluted in PBS, and plated on LB agar plates in triplicate. The plates were grown at 37 °C overnight before counting and determining CFU/ml. The average results were plotted, and a one-way ANOVA with Tukey’s multiple comparison test was used to determine whether the difference was significant. The OD matched samples were then further diluted in HEKblue cell culture media prior to the TLR4 activation assay. **Figure S4.** Frame shift in wzE within the pTarget recombination construct Δ*wzzB*(*wzD-wzE).* Chromatogram of sequence viewed in Chromas software (Technelysium Pty Ltd., Australia) with the base after deletion highlighted. Alignment of the pTarget recombination construct sequence (top) against the *S. pneumoniae* TIGR4 genome sequence (bottom) with ATG start codon of wzE in green. A thymine (T) is missing from the pTarget recombination construct sequence which causes a frame shift in the open reading frame of wzE. **Figure 5.** Conjugation of *C. jejuni* heptasachcaride to AcrA. Overnight culture was used to seed 2YP broth to OD_600_ of 0.03. After 4 h 1 mM IPTG was added to W311B pPgl::pglB pWA2. After 24 h growth at 28 °C cells were harvested, matched to OD_600_ 20 and lysed with FastPrep homogenizer. Fifteen µl lysate was loaded on a Bolt 4–12% bis-tris gel with MOPS buffer. After transfer to nitrocellulose membrane protein and glycan were detected using mouse anti-His monoclonal antibody (Abcam, UK) and HR6 antiserum (S. Amber and M. Aebi, unpublished data) respectively. Secondary goat anti-rabbit IgG IRDye 800 and goat anti-mouse IgG IRDye 680 conjugates were used tp generate fluorescent signal which was detected using an Odyssey LI-COR detection system (LI-COR Biosciences UK Ltd.). Green channel is for Glycan, Red for protein. All strains contained pACYCPgl::pglB and pWA2. CLM24 does not contain a functional PglB transferase so only unclycosylated AcrA is visible. W311B contains chromosomally integrated *pglB* under a P_tac_ promoter and the remaining strains contain integrated *pglB* under an Anderson X10 promoter. *M* marker (PageRuler plus prestained protein ladder—Fisher). AcrA runs at 40 KDa.

## Data Availability

All data generated or analysed during this study are included in this published article and its additional files.
