# Supplementary material for: Engineering a suite of E. coli strains for enhanced expression of bacterial polysaccharides and glycoconjugate vaccines
Source: Microb Cell Fact. 2022 Apr 21;21:66. doi: 10.1186/s12934-022-01792-7 (PMC9026721; doi:10.1186/s12934-022-01792-7)
Supplement: Supplementary file 1 — Additional file 1: Table S1. Plasmids used in this study. Table S2. Strains used in this study. Table S3. N20 specific sequence of gRNA and protospacer adjacent motif (PAM). Table S4. Primers for mutant confirmation. Table S5. 2 way ANOVA multiple comparisons for TLR4 activation assay. Figure S1. PCR Verification of mutations within strains. Overnight cultures of each strain were lysed using Chelex 100 (BioRad). PCR was performed with Platinum Green Hot start PCR mastermix (Invitrogen), in 20 µl reaction, according to manufacturer’s instructions, using Tm determined by NEB Tm calculator. Ten µl was run on an agarose gel alongside Hyperladder 1 Kb marker (Bioline) and visualized with gelRed nucleic acid stain (biotium). M marker; B negative control no template PCR. Figure S2. Growth curve of all strains. Overnight cultures of all strains were used to inoculate fresh LB broth to an OD600 of 0.03. Cultures were incubated at 37 °C with shaking and OD600 measurements taken at regular intervals with a final reading taken at 24 h. Strains did not contain plasmids. Strains depicted in red contain pglB and grow slightly slower than those without and reach a slightly lower final OD600. W3110 is included as a wild type comparison. The experiment was performed in triplicate and the data shown are mean values with error bars depicting standard error of the mean. Figure S3. Colony forming units for TLR4 activation assay. Overnight cultures of W3110, Raptor and ClearColi strains were matched for OD600 value, serially diluted in PBS, and plated on LB agar plates in triplicate. The plates were grown at 37 °C overnight before counting and determining CFU/ml. The average results were plotted, and a one-way ANOVA with Tukey’s multiple comparison test was used to determine whether the difference was significant. The OD matched samples were then further diluted in HEKblue cell culture media prior to the TLR4 activation assay. Figure S4. Frame shift in wzE within the pTarget recombination [file 12934_2022_1792_MOESM1_ESM.docx]

Additional file

# Additional Tables

Table S1: Plasmids used in this study

| **Plasmid** | **Characteristics** | **Origin of Replication** | **Source or Reference** |
| --- | --- | --- | --- |
| pB | pBBR1MCS-3 (Tc^R^) | pBBR1 | (Kovach et al. 1995) |
| pB4 | *S. pneumoniae* serotype 4 capsule locus (*wciI-fnlC*) pBBR1MCS-3 (Tc^R^) | pBBR1 | (Kay et al. 2016) |
| pMAF12 | *gne* *C. jejuni* 81116 pEXT21 (Sp^R^) | incW | M. Feldman  (Dykxhoorn et al. 1996) |
| pEXT21:wzDE | *wzD-wzE* *S. pneumoniae* TIGR4 pEXT21 (Sp^R^) | incW | This study |
| pEXT22:PglB | P_tac_ *pglB* *C. jejuni* 81116 pEXT22 (Km^R^) | R100 | (Samaras et al. 2021) |
| pTarget | sgRNA (Ap^R^) | pMB1 | (Jiang et al. 2015) |
| pCas | P*_araB_*-Red *lacI*^q^ P*_trc_*-sgRNA-pMB1 (Km^R^) | repA101(Ts) | (Jiang et al. 2015) |
| pACYCPgl::pglB | pACYCpglB::Km. *C. jejuni* strain 81116 *pgl* locus (*pglA-wlaB*). *pglB* interrupted with EZ::Tn transposon system. (Cm^R^ Km^R^) | p15A | (Linton et al. 2005) |
| pWA2 | *C. jejuni* *acrA* with a *pelB* signal sequence and His6-tag. (Ap^R^) | pBR322 | (Feldman et al. 2005) |

Table S2: Strains used in this study

| Strain | Characteristics | Source or Reference |
| --- | --- | --- |
| ClearColi BL21 | F- ompT hsdSB (rB- mB-) gal dcm lon λ(DE3 [lacI lacUV5-T7 gene 1 ind1 sam7 nin5]) msbA148 ΔgutQ ΔkdsD ΔlpxL ΔlpxM ΔpagP ΔlpxP ΔeptA | Lucigen Corporation, USA |
| *E. coli* W3110 | F-lambda-IN(*rrnD-rrnE*)1 rph-1 | (Bachmann 1996) |
| *E. coli* CLM24 | W3110 Δ*waaL* | (Feldman et al. 2005) |
| *E. coli* CLM37 | W3110 Δ*wecA* | (Linton et al. 2005) |
| *E. coli* W311B | W3110 ::P_tac_ *cjpglB* | (Herbert et al. 2018) |
| Raptor | W3110 Δ*lpxM* | This Study |
| Falcon | W3110 Δ*lpxM*, *ΔwecA-wzzE*(*gne*) | This Study; (Samaras et al. 2021) |
| Hobby | W3110 Δ*lpxM*, *ΔwecA-wzzE*(*gne*), Δ*waaL* | This Study |
| Crow | W3110 Δ*lpxM*, Δ*waaL* | This Study |
| Peregrine | W3110 Δ*lpxM*, Δ*wecA-wzzE*(*gne*), Δ*wzzB*(*wzD-wzE*) | This Study |
| Sparrowhawk | W3110 Δ*lpxM*, Δ*wecA-wzzE*(*gne*), Δ*waaL*, Δ*wzzB*(*wzD-wzE*) | This Study |
| Osprey | W3110 Δ*lpxM*, Δ*wecA-wzzE*(*gne*), Δ*waaL*, Igs#*atpI-gidB*(*CjpglB*) | This Study |
| Merlin | W3110 Δ*lpxM*, Δ*wecA-wzzE*(*gne*), Δ*waaL*, Δ*wzzB*(*wzD-wzE*), Igs#*atpI-gidB*(*CjpglB*) | This Study |
| Eagle | W3110 Δ*lpxM*, Δ*wecA-wzzE*(*gne*), Igs#*atpI-gidB*(*CjpglB*) | This Study |
| Kestrel | W3110 Δ*lpxM*, Δ*wecA-wzzE*(*gne*), Δ*wzzB*(*wzD-wzE*), Igs#*atpI-gidB*(*CjpglB*) | This Study |
| Raven | W3110 Δ*lpxM*, Δ*waaL*, Igs#*atpI-gidB*(*CjpglB*) | This Study |

Table S3: N20 specific sequence of gRNA and protospacer adjacent motif (PAM)

CRISPR gRNA cas9 specific portion of gRNA:

GTTTTAGAGCTAGAAATAGCAAGTTAAAATAAGGCTAGTCCGTTATCAACTTGAAAAAGTGGCACCGAGTCGGTGCTTTTTTT

| Target | N20 + PAM^1^ |
| --- | --- |
| *lpxM* | CTAACAGATCATCCATCGGTGGG |
| *wecA* | GATTGATACCTCTCGTTGGGGGG |
| *waaL* | GAACAGGAGTAGGGTTGCTCTGG |
| *wzzB* | CAAGATACGTTGTTCCTTTTGGG |
| *wzD* | TGACGTGACAACACTGGAGGAGG |
| *atpB* | TTATTAAAAATGTCAATGGGTGG |

^1^PAM sequence is shown in red. These bases do not form part of the gRNA.

Table S4: Primers for mutant confirmation

| Primer name | Sequence 5`-3` | use | Expected product size |
| --- | --- | --- | --- |
| lpxmOL | atcgtaacggtactggtctg | Confirm *lpxM* deletion | WT = 2609 bp  Mutant = 1613 bp |
| lpxmOR | ttgattggcgtagattacct |  |  |
| rfeOL | ccggttctaaaatggacgaa | Confirm *wecA* deletion | WT = 3402 bp  Mutant = 2242 bp |
| wzzEOR | aaactctcacgcctgtgacc |  |  |
| RfaLOL | gcacagcattttctgacgaa | Confirm *waaL* deletion | WT = 2513 bp  Mutant = 1478 bp |
| RfaLOR | gcatctgcgggatttacagt |  |  |
| CldOL | agcaaaatatcccgatgctg | Confirm *wzzB* deletion | WT = 2414 bp  Mutant = 3257 bp |
| CldOR | ggacttatgccggtgattgt |  |  |
| atpBOL | aaccaggaacaacagaccca | Confirm *pglB* insertion | WT = 1561 bp  Mutant = 3427 bp |
| gidBOR | tccactctctatcgtgcgtc |  |  |

Table S5: 2 way ANOVA multiple comparisons for TLR4 activation assay

| Tukey's multiple comparisons test | Mean Diff. | 95.00% CI of diff. | Below threshold? | Summary | Adjusted P Value |
| --- | --- | --- | --- | --- | --- |
|  |  |  |  |  |  |
| 10^5^:W3110 vs. 10^5^:Raptor | 0.6216 | 0.5490 to 0.6942 | Yes | **** | <0.0001 |
| 10^5^:W3110 vs. 10^5^:ClearColi | 1.12 | 1.048 to 1.193 | Yes | **** | <0.0001 |
| 10^5^:W3110 vs. 10^4^:W3110 | 0.3223 | 0.2497 to 0.3949 | Yes | **** | <0.0001 |
| 10^5^:W3110 vs. 10^4^:Raptor | 1.075 | 1.002 to 1.147 | Yes | **** | <0.0001 |
| 10^5^:W3110 vs. 10^4^:ClearColi | 1.221 | 1.148 to 1.293 | Yes | **** | <0.0001 |
| 10^5^:W3110 vs. 10^3^:W3110 | 0.9514 | 0.8788 to 1.024 | Yes | **** | <0.0001 |
| 10^5^:W3110 vs. 10^3^:Raptor | 1.217 | 1.144 to 1.289 | Yes | **** | <0.0001 |
| 10^5^:W3110 vs. 10^3^:ClearColi | 1.231 | 1.159 to 1.304 | Yes | **** | <0.0001 |
| 10^5^:Raptor vs. 10^5^:ClearColi | 0.4987 | 0.4261 to 0.5713 | Yes | **** | <0.0001 |
| 10^5^:Raptor vs. 10^4^:W3110 | -0.2993 | -0.3719 to -0.2267 | Yes | **** | <0.0001 |
| 10^5^:Raptor vs. 10^4^:Raptor | 0.453 | 0.3805 to 0.5256 | Yes | **** | <0.0001 |
| 10^5^:Raptor vs. 10^4^:ClearColi | 0.5989 | 0.5264 to 0.6715 | Yes | **** | <0.0001 |
| 10^5^:Raptor vs. 10^3^:W3110 | 0.3298 | 0.2572 to 0.4023 | Yes | **** | <0.0001 |
| 10^5^:Raptor vs. 10^3^:Raptor | 0.595 | 0.5224 to 0.6676 | Yes | **** | <0.0001 |
| 10^5^:Raptor vs. 10^3^:ClearColi | 0.6097 | 0.5371 to 0.6822 | Yes | **** | <0.0001 |
| 10^5^:ClearColi vs. 10^4^:W3110 | -0.798 | -0.8706 to -0.7254 | Yes | **** | <0.0001 |
| 10^5^:ClearColi vs. 10^4^:Raptor | -0.04567 | -0.1182 to 0.02689 | No | ns | 0.4419 |
| 10^5^:ClearColi vs. 10^4^:ClearColi | 0.1002 | 0.02768 to 0.1728 | Yes | ** | 0.0033 |
| 10^5^:ClearColi vs. 10^3^:W3110 | -0.1689 | -0.2415 to -0.09638 | Yes | **** | <0.0001 |
| 10^5^:ClearColi vs. 10^3^:Raptor | 0.0963 | 0.02374 to 0.1689 | Yes | ** | 0.0048 |
| 10^5^:ClearColi vs. 10^3^:ClearColi | 0.111 | 0.03841 to 0.1835 | Yes | ** | 0.0011 |
| 10^4^:W3110 vs. 10^4^:Raptor | 0.7523 | 0.6798 to 0.8249 | Yes | **** | <0.0001 |
| 10^4^:W3110 vs. 10^4^:ClearColi | 0.8982 | 0.8257 to 0.9708 | Yes | **** | <0.0001 |
| 10^4^:W3110 vs. 10^3^:W3110 | 0.6291 | 0.5565 to 0.7016 | Yes | **** | <0.0001 |
| 10^4^:W3110 vs. 10^3^:Raptor | 0.8943 | 0.8217 to 0.9669 | Yes | **** | <0.0001 |
| 10^4^:W3110 vs. 10^3^:ClearColi | 0.909 | 0.8364 to 0.9815 | Yes | **** | <0.0001 |
| 10^4^:Raptor vs. 10^4^:ClearColi | 0.1459 | 0.07334 to 0.2185 | Yes | **** | <0.0001 |
| 10^4^:Raptor vs. 10^3^:W3110 | -0.1233 | -0.1958 to -0.05071 | Yes | *** | 0.0003 |
| 10^4^:Raptor vs. 10^3^:Raptor | 0.142 | 0.06941 to 0.2145 | Yes | **** | <0.0001 |
| 10^4^:Raptor vs. 10^3^:ClearColi | 0.1566 | 0.08408 to 0.2292 | Yes | **** | <0.0001 |
| 10^4^:ClearColi vs. 10^3^:W3110 | -0.2692 | -0.3417 to -0.1966 | Yes | **** | <0.0001 |
| 10^4^:ClearColi vs. 10^3^:Raptor | -0.00393 | -0.07649 to 0.06862 | No | ns | >0.9999 |
| 10^4^:ClearColi vs. 10^3^:ClearColi | 0.01073 | -0.06182 to 0.08329 | No | ns | 0.9998 |
| 10^3^:W3110 vs. 10^3^:Raptor | 0.2652 | 0.1927 to 0.3378 | Yes | **** | <0.0001 |
| 10^3^:W3110 vs. 10^3^:ClearColi | 0.2799 | 0.2073 to 0.3525 | Yes | **** | <0.0001 |
| 10^3^:Raptor vs. 10^3^:ClearColi | 0.01467 | -0.05789 to 0.08722 | No | ns | 0.998 |

# Additional Data

Sequences for Recombination templates:

Upstream flank

Downstream Flank

Promoter

Inserted Gene

Δ*lpxM*:

caccacacagaggtgttgatttcgccatgccgcagggtacgccagtgctttcagtgggtgacggtgaagtggtggttgccaaacgcagtggcgcagcaggttattatgtggctattcgtcatggtcgcagctacaccacgcgttatatgcacttgcgcaagattctggtgaaaccgggacagaaggtgaaacgtggcgaccgtatcgcgctttccggtaataccggacgttcaaccgggccgcatctgcactatgaagtatggataaaccagcaggccgtaaacccgctgacggcaaaactgccgcgtaccgaagggctgaccggctccgatcgtcgcgaattcctggcgcaggccaaagagattgtgccgcagctacggtttgattaattaacatccattcgcagccggtacgcagtcagtaccggctttttttatttggtgcggggcaagttgcgccgctacactatcaccagattgatttttgccttatccgaaactggaaaagcccttcgcctgatgataagttcaagtttgcttcagaatattcgaaatctgttgaactatcattgaactgtaggccggatgtggcgttttcgccgcatccggcaacgtacttactctaccgttaaaatacgcgtggtattagtagaacccacggtactcatcacgtcgccctgggtgacaatcaccaggtcaccagacatcaagtaacctttatcgcgcagcagattaaccgcttcgctggcagctgctacgccgtcattagcgctatcaaagtgcaccggcgtaacgccacgatagagagcagtcaggttcagcgtacgttcatggcgcgacatggcgaaaattggcagaccagagctgatacgggaggtcatcagcgcggtacgacccgattcggtcatggtgatgatcgccgtaacgcctttcaggtggttagctgcgtacattgctgacatggcaatagcttcttccacattgtcgaactgaacgtccagacggtgtttagaaacgttgatgctcgggattttttccgcacccaggcaaacgcgcgccatggctgcaacggtttctgacggatactgcccagcggcagtttctgcagacagcatcacagcgtcagtaccat

Δ*wecA-wzzE*(*gne*):

aaaggtacaggcaacatggaactgcacctctctcgtaagatcgctgaaaaacgcgtcttcccggctatcgactacaaccgttctggtacccgtaaagaagagctgctcacgactcaggaagaactgcagaaaatgtggatcctgcgcaaaatcattcacccgatgggcgaaatcgatgcaatggaattcctcattaataaactggcaatgaccaagaccaatgacgatttcttcgaaatgatgaaacgctcataaatttgtcttatgccaaaaacgccacgtgtttacgtggcgttttgcttttatatctgtaatcttaatgccgcgctggcgatgttaggaaaattcctggaatttgctggcatgttatgcaatttgcatatcaaatggttaatttttgcacaggactggtgggtttggaacggactttcccttctgaataaaggtcttcgtggttatacttctgctaataattttctctgagagcatgcattgtgaatatgaaaattcttattagcggtggtgcaggttatataggttctcatactttaagacaatttttaaaaacagatcatgaatttgtgttttagataatctttctaagggttctaaaatcgcaatagaagatttgcaaaaaataagaacttttaaattttttgaacaagatttaagtgattttcaaggcgtaaaagcattgtttgagagagaaaaatttgacgctattgtgcattttgcagcgagcattgaagtttttgaaagtatgcaaaaccctttaaagtattatatgaataacactgttaatacgacaaatctcatcgaaacttgtttgcaaactggagtgaataaatttatattttcttcaacggcagccacttatggcgaaccacaaactcccgttgtgagcgaaacaagtcctttagcacctattaatccttatgggcgtagtaagcttatgagcgaagaggttttgcgtgatgcaagtatggcaaatcctgaatttaagcattgtattttaagatattttaatgttgcaggtgcttgcatggattatactttaggacaacgctatccaaaagcgactttgcttataaaagttgcagctgaatgtgccgcaggaaaacgtaataaacttttcatatttggcgatgattatgatacaaaagatggcacttgcataagagattttatccatgtggatgatatttcaagtgcgcatttatcggctttggattatttaaaagagaatgaaagcaatgtttttaatgtaggttatggacatggttttagcgtaaaagaagtgattgaagcgatgaaaaaagttagcggagtggattttaaagtagaacttgccccacgccgtgcgggtgatcctagtgtattgatttctgatgcaagtaaaatcagaaatcttacttcttggcagcctaaatatgatgatttagggcttatttgtaaatctgcttttgattgggaaaaacagtgctaatgctcgaaatagcaacactgctgcggtgagcgcaaaggcgctcgccgcttattcgaagagaatcgatgtgaaagtactgactgtatttggtacgcgcccggaagccatcaagatggcgccgttggtgcatgcgttggcaaaagatcctttttttgaggctaaagtttgcgtcactgcgcagcatcgggagatgctcgatcaggtgctgaaactcttttccattgtacctgactacgatctcaacataatgcagccaggacagggcctgacagagataacctgtcggattctggaagggctaaaacctattcttgccgagttcaaaccagacgtcgtgctggttcacggcgatacgacgacgacgctggcaaccagcctggcggcgttttatcagcgtattcctgttggtcacgttgaggctggtctgcgcacgggcgatctctattcgccgtggccggaagaggctaaccgtacattgaccgggcatctggcgatgtatcacttctctccaaccgaaacttcccggcaaaacttgctgcgtgaaaacgttgcggatagccgaatcttcattaccggtaatacagtcattgatgcactgttatgggtg

Δ*waaL*:

cgaatatgccgtatttcttcatgcgacgacccgtgatgataaacactggccggaagaacactggcgagaattgattggtttactggctgattcaggaatacggattaaacttccgtggggcgcgccgcatgaggaagaacgggcgaaacgactggcggaaggatttgcttatgttgaagtattgccgaagatgagtctggaaggcgttgcccgcgtgctggccggggctaaatttgtagtgtcggtggatacggggttaagccatttaacggcggcactggatagacccaatatcacggtttatggaccaaccgatccgggattaattggtgggtatgggaagaatcagatggtatgtagggctccaagagaaaatttaattaacctcaacagtcaagcagttttggaaaagttatcatcattataaaggtaaaacatgctaacatcctttaaacttcattcattgaaaccttacactctgaaatcatcaatgattttagagataataacttatatattatgttttttttcaatgataattgcattcgtcgatgattggactcagtgatgtgatcatatgggcacgcagcattccaattatcattatatccgctatagtcctcttactcgtcattaataatcgtaacaatacaattaattaagaataaacaagtttaagaagtgagttaaaactcacttcttatctatacaacttaatctctttactgaattagttaaagtttcagtatcgatatcttttaccgtatatgttggggatactatttgtatagatttatgatgattcggggaccaaattaaatgcgagggatattccggagtacgtgaattagggtaaaatgcaagcgttggtttatgataagccgcagcgatatgaacaagagctgtatcaacagaaatgacaaaatcactatacttggtcaacgcaacggtataaataaactcatcaaatggtagtgtttcgatttcaagtattggtattgtcaataaatcttgcggtaacccggtaaatataatccgataattttcaaagtgtgttttcacttcttgatatataacttttatttgctcaaacgtaagacggcatattttttttgcacctaatggattaataatgactattcttgtatctccaataaattcttttattttatcttcaacatctactggtaaatgcaagtcgtagttggtggaaaatttaccctcaccataaatatgcttcagtatttctattgctcgtgtactcatatgttcttttaaacattcatcgtggggatggtaaaatgaatagtaacgcttataccaatgatcaaaacccagaatg

Δ*wzzB*(*wzD-wzE*):

ctgccgaaagataccaagcagttactggcgaactaccagtctgtgccgaataacctgatctcggcaattgtcgatgctaaccgcacgcgtaaagattttattgccgatgccattttgtcacgcaagccgcaagtggtgggtatttatcgtctgattatgaagagcggttcagataacttccgtgcgtcttctattcaggggattatgaaacgtatcaaggcgaaaggtgttgaagtgatcatctacgagccagtgatgaaagaagactcattcttcaactctcgcctggaacgtgatctcgccaccttcaaacaacaagccgacgtcattatctctaaccgaatggcagaagagcttaaggatgtggcagataaggtatacacccgcgatctctttggcagcgactaacatcttgttatcagggctatttacgccctgattgtcttttgttacttacacaacaattcattatttttatcacttatcctatagcattcacgaggattatcgctaaactatgcggacttggaaatttccgtcagttagggtaatgatgagagtaccacaattcagcaaattgtgaacatcatcacgttcatctttccctggttgccaatggcccattttcctgtcagtaacgagaaggtcgcgaattcaggcgctttttagactggtcgtaatgaagggagaccacaacggtttccctctagaaataattttctttaactttaagaaggagatatatccatgatgaaagaacaaaatacgatagaaatcgatgtatttcaattagttaaaagcttgtggaaacgcaagctaatgattttaatagtggcacttgtgacaggtgcgggggcttttgcatatagcacttttattgttaagccagaatatacgagtaccacgcgaatttacgtagtgaatcgcaatcaaggagacaagccggggttgacaaatcaggatttgcaggcaggaacttatctggtaaaagactaccgtgagattatcctttcgcaggatgttttggaggaagttgtttctgatttgaaactagatttgacgccaaaaggtttggctaataaaattaaagtgacagtaccagttgatacccgtattgtctctatttcagttaatgatcgagttcctgaagaggcaagccgtatcgctaactctttgagagaagtagctgctcaaaaaattatcagtattactcgtgtttctgacgtgacaacactggaggaggcaaggccggcgatatccccgtcttcgccaaatattaaacgcaatacactaattggttttttggcaggggtgattggaaccagtgttatagttcttcatcttgaacttttggatactcgtgtgaaacgtccggaagatatcgaaaatacattgcagatgacacttttgggagttgtgccaaacttgggtaagttgaaataggagagaaggatgccgacattagaaatagcacaaaaaaaactggagttcattaagaaggcagaagaatattacaatgccttgtgtacaaatatacagttgagcggagataaactaaaagtaatttccgttacttctgttaaccctggggaaggaaaaacaactacttccataaatatagcatggtcgtttgcgcgtgcaggctataaaactcttttgatcgatggcgatactcgaaattcagttatgttaggagtttttaaatctcgtgaaaaaattacagggctaacagaatttttatctgggacagctgatttatctcacggtttatgtgatacaaatattgaaaatttatttgtagttcaatcgggatctgtatcaccaaaccctacagccttgttacaaagtaaaaattttaatgatatgattgaaacattgcgtaaatattttgattatatcattattgatacaccgcctattggaattgttattgatgcggcaattatcactcaaaagtgtgatgcgtccatcttggtaacagcaacaggtgaggcgaataaacgtgatatccaaaaagcgaaacaacaattaaaacaaacagggaaactgttcctaggagttgttttaaataaattggatatctcggttaataagtatggagtttacggttcctatggaaattatggtaaaaaataacgttggtgttctcgccaaactactatcagactcgtcaaaacctgctggatatcgaaagcttaaaagttgatgatcttgatattcatgcttaccgttatgtgatgaaaccgatgttacctattcgtcgtgatagcccgaaaaaggcaattaccttgattctggcggtgctgctgggtggcatggttggcgcggggattgtgctggggcgtaatgctctacgcaattacaacgcgaagtaatcttttcggttttaaagaaaaagggcagggtggtgacaccttgcccgtttttttgccggatgcggcaacaatatcgcatccgcttaccccgcaactcactgatgccgtttacgcaggttctcaattaccgtcgttaaatccagcccctgatcctgcaacaacaccagcaggtgatacatcaaatcagacgcctcgttggtcagctcaaagcggtcatgtaccgttgctgccagcgcggtttccacgccttcttcacccactttctgcgcaatgcgtttggtgccgctggcatacagtttggcggtgtaggag

Igs#*atpI-gidB*(*CjpglB*):

Ccttttaccctttgttgttaattacagccggtgccagtatctgaaccaccagcaccaaaacccacgtaacgatcagcggcaagaataccgcctttaaaaccgccaacgccaccaccagtaacaccaacatcgccagaactttgaaagcttcgccaaatgcgaatgtccaggccacccggcctttcgctggtgtatgcgcctggtgacgccaggcaaatatcataaacaaaacgttaggcagaaagactgccaggcccccgcttattgcagagacgccccagaaggggtctttgaggctgaacagcaatccacttgctatcaccaccagtaactgaacgagcagaagcttccgagcaacgtttcgactcacgagcgacacagacatcacgtttttcactcctgctcccttcgaggtatgccgcgtgtcgtataaaactttctttaaggcttagagtcaagcatcaaaaagcggtcaaattatacggtgcgcccccgtgatttcaaacaataagtatttatggctagctcagtcctaggtacaatgctagcaggaggaaaaaatgttgaaaaaagagtatttaaaaaacccttatttagttttgtttgcgatgattgtattagcttatgtttttagtgtattttgcaggttttattgggtttggtgggcaagtgagtttaacgagtattttttcaataatcaattaatgatcatttcaaacgatggctatgcttttgctgagggcgcaagagatatgatagcaggttttcatcagcctaatgatttgagttattatggatcttctttatctacgcttacttattggctttataaaatcacacctttttcttttgaaagtatcattttatatatgagtacttttttatcttctttggtggtgattcctattattttactagctaatgaatacaaacgccctttaatgggctttgtagctgctcttttagcaagtgtagcaaacagttattataatcgcactatgagtgggtattatgatacggatatgctggtaattgttttacctatgtttattttattttttatggtaagaatgattttaaaaaaagactttttttcattgattgccttgccattatttataggaatttatctttggtggtatccttcaagttatactttaaatgtagctttaattggactttttttaatttatacacttatttttcatagaaaagaaaagattttttatatagctgtgattttgtcttctcttactctttcaaatatagcatggttttatcaaagtgccattatagtaatactttttgctttatttgctttagagcaaaaacgcttaaattttatgattataggaattttaggtagtgcaactttgatatttttgattttaagtggtggggttgatcccatactttatcagcttaaattttatatttttagaagcgatgaaagtgcgaatttaacacagggctttatgtattttaatgttaatcaaaccatacaagaagttgaaaatgtagattttagcgaatttatgcgaagaattagtggtagtgaaattgttttcttgttttctttgtttggttttgtatggcttttgagaaaacataaaagtatgattatggctttacctatattggtgcttgggtttttagccttaaaaggaggacttagatttaccatttattctgtacctgtaatggctttaggatttggttttttattgagcgagtttaaggctatattggttaaaaaatatagccaattaacttcaaatgtttgtattgtttttgcaactattttgactttggctccagtatttatccatatttacaactataaagcgccaacagttttttctcaaaatgaagcatcattattaaatcaattaaaaaatatagccaatagagaagattatgtggtaacttggtgggattatggttatcctgtgcgttattatagcgatgtgaaaactttagtagatggtggaaagcatttaggtaaggataattttttcccttctttttctttaagtaaagatgaacaagctgcagctaatatggcaagacttagtgtagaatatacagaaaaaagcttttatgctccgcaaaatgatattttaaaatcagacattttacaagccatgatgaaagattataatcaaagcaatgtggatttatttctagcttcattatcaaaacctgattttaaaatcgatacaccaaaaactcgtgatatttatctttatatgcccgctagaatgtctttgattttttctacggtggctagtttttcttttattaatttagatacaggagttttggataaaccttttacctttagcacagcttatccacttgatgttaaaaatggagaaatttatcttagcaacggagtggttttaagcgatgattttagaagttttaaaataggtgataatgtggtttctgtaaatagtatcgtagagattaattctattaaacaaggtgaatacaaaatcactccaatcgatgataaggctcagttttatattttttatttaaaggatagtgctattccttacgcacaatttattttaatggataaaaccatgtttaatagtgcttatgtgcaaatgttttttttgggaaattatgataagaatttatttgacttggtgattaattctagagatgctaaagtttttaaacttaaaatttaaatattttcactaatgacttattttctgcttaccaaaaaaagccacgttatcttgttgatgcaaaagagtgaacgtggcgttaaatgtaaccagttatatcagtagaaaacctggttgttgttaacagtctaaccggtcaattttttatgatttttttgataaaaattaaattttatttgctttaatcaccaccagatgacgttcgccatccagggctggaacctgaagtttaaccactgattcgacctgatattcttcgggcaacaaagcgatttcatcttccggcatttgccctttcagcgcgtagaaacggccttgctcaccaggaagatggtggcaccagctcaccatatcgttcagagaggcaaaagcgcggctaattacgccatcaaatggcggctctgaaggaaactcttctaccctgctctgtactggttcaatattctccagtttaagctcatgttgcacctgacgaaggaaacgcacgcgtttaccaaggct

*wzE* patch:

gaggag changed to gaagaa to remove gRNA cut site but maintain amino acid sequence. Thymine (T) added back to sequence to repair frame shift in *wzE* is shown in black.

tgctttttttcgtagtgaatcgcaatcaaggagacaagccggggttgacaaatcaggatttgcaggcaggaacttatctggtaaaagactaccgtgagattatcctttcgcaggatgttttggaggaagttgtttctgatttgaaactagatttgacgccaaaaggtttggctaataaaattaaagtgacagtaccagttgatacccgtattgtctctatttcagttaatgatcgagttcctgaagaggcaagccgtatcgctaactctttgagagaagtagctgctcaaaaaattatcagtattactcgtgtttctgacgtgacaacactggaagaagcaaggccggcgatatccccgtcttcgccaaatattaaacgcaatacactaattggttttttggcaggggtgattggaaccagtgttatagttcttcatcttgaacttttggatactcgtgtgaaacgtccggaagatatcgaaaatacattgcagatgacacttttgggagttgtgccaaacttgggtaagttgaaataggagagaaggatgccgacattagaaatagcacaaaaaaaactggagttcattaagaaggcagaagaatattacaatgccttgtgtacaaatatacagttgagcggagataaactaaaagtaatttccgttacttctgttaaccctggggaaggaaaaacaactacttccataaatatagcatggtcgtttgcgcgtgcaggctataaaactcttttgatcgatggcgatactcgaaattcagttatgttaggagtttttaaatctcgtgaaaaaattacagggctaacagaatttttatctgggacagctgatttatctcacggtgcagaagcttagatctatt

# Additional Figures

**PCR verification of mutants**


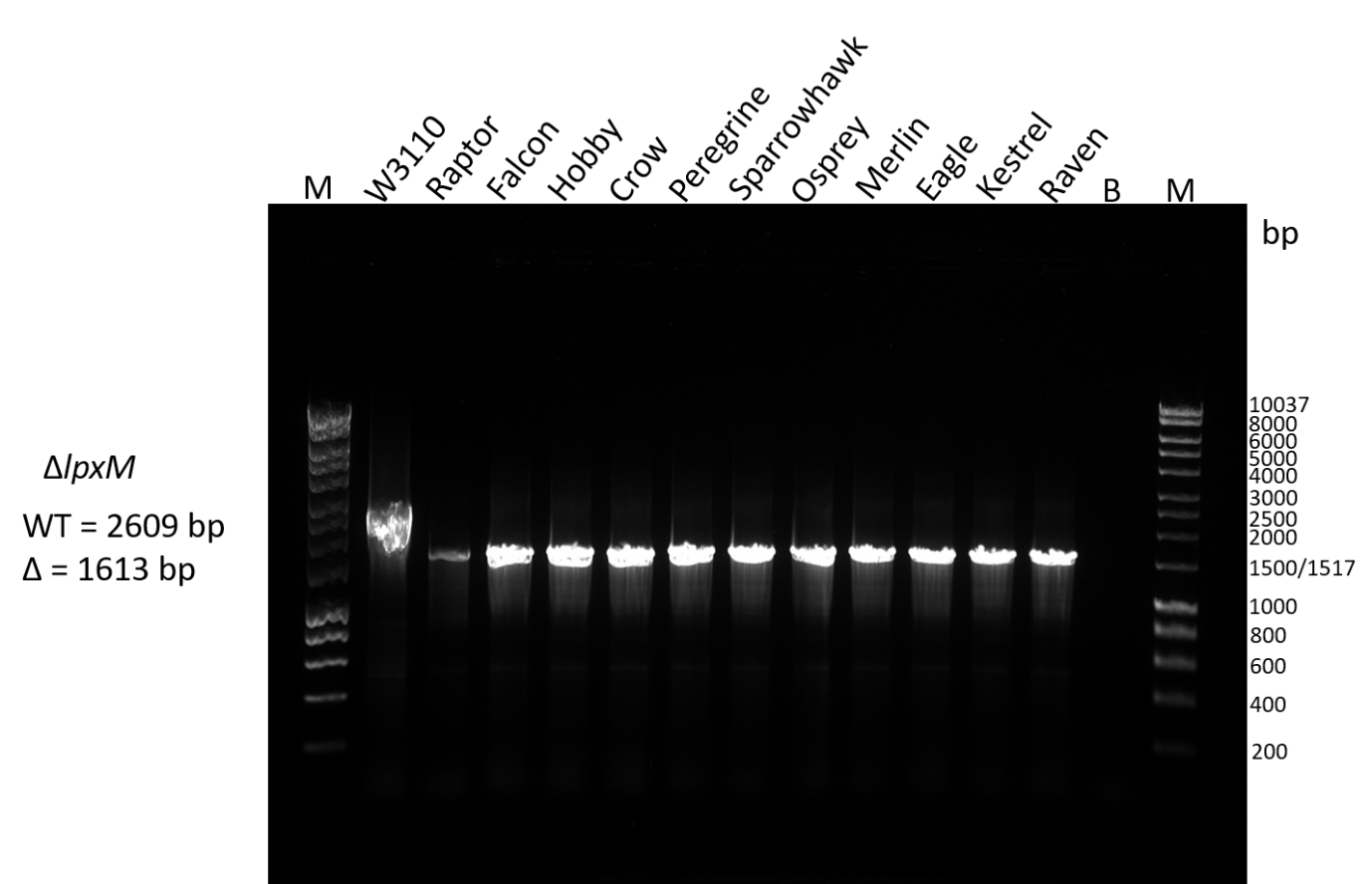


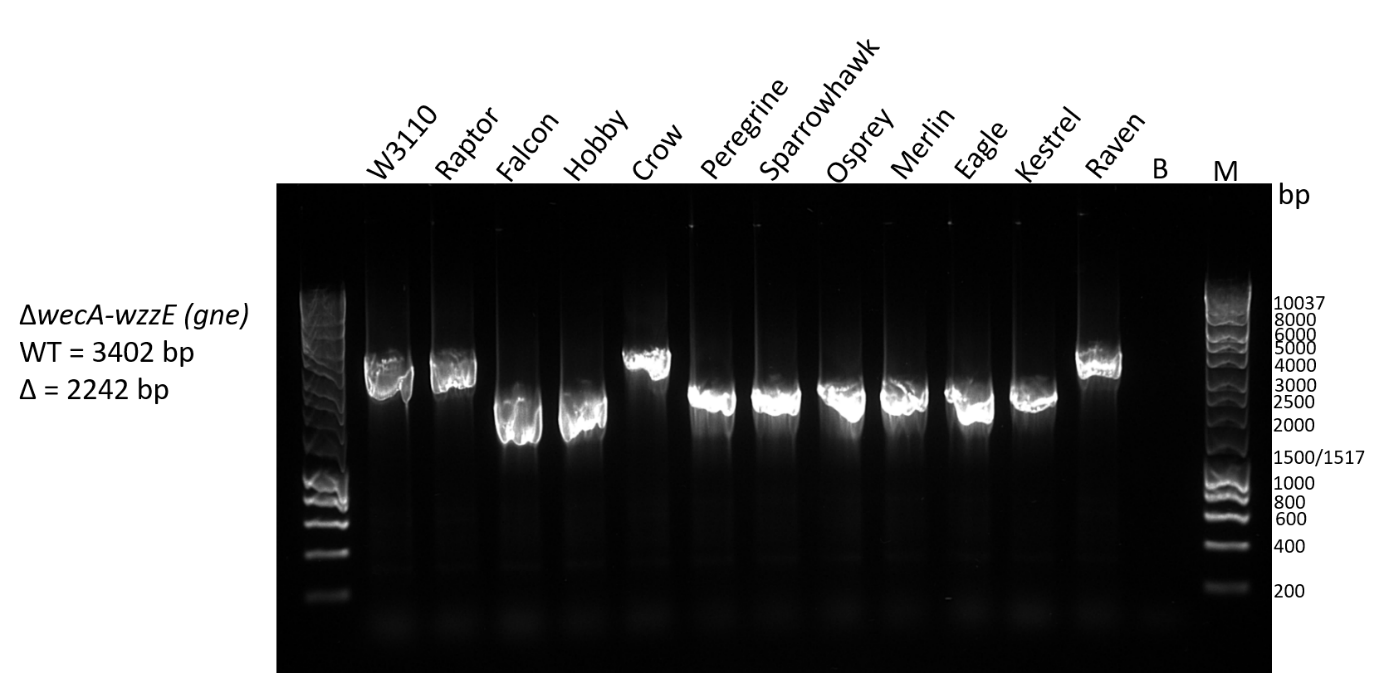


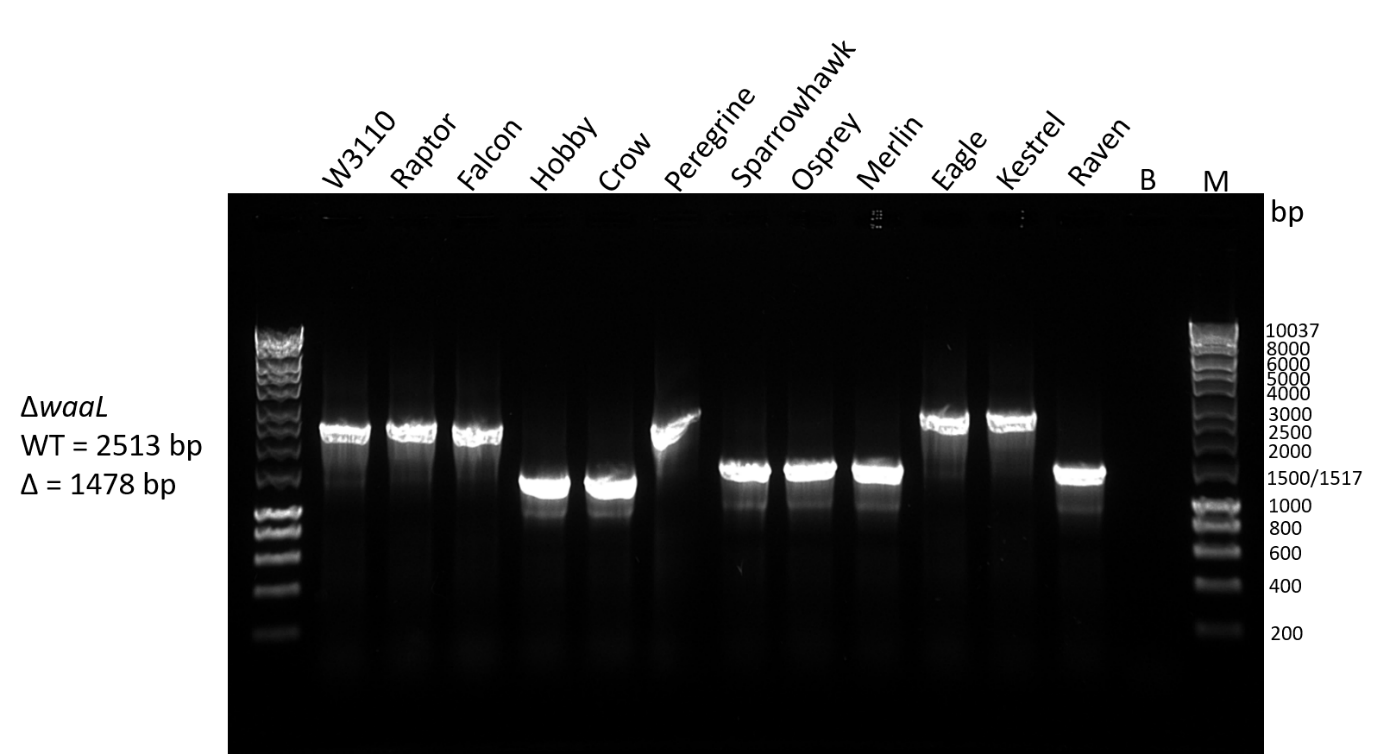


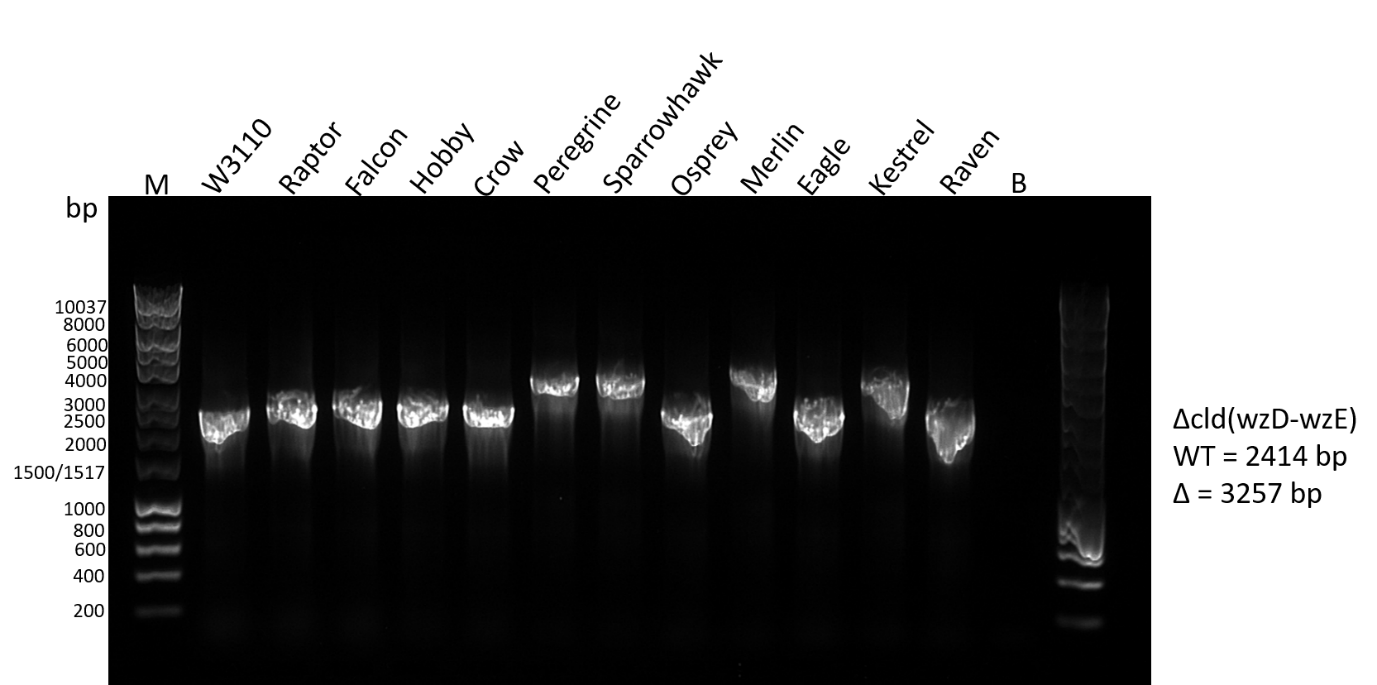


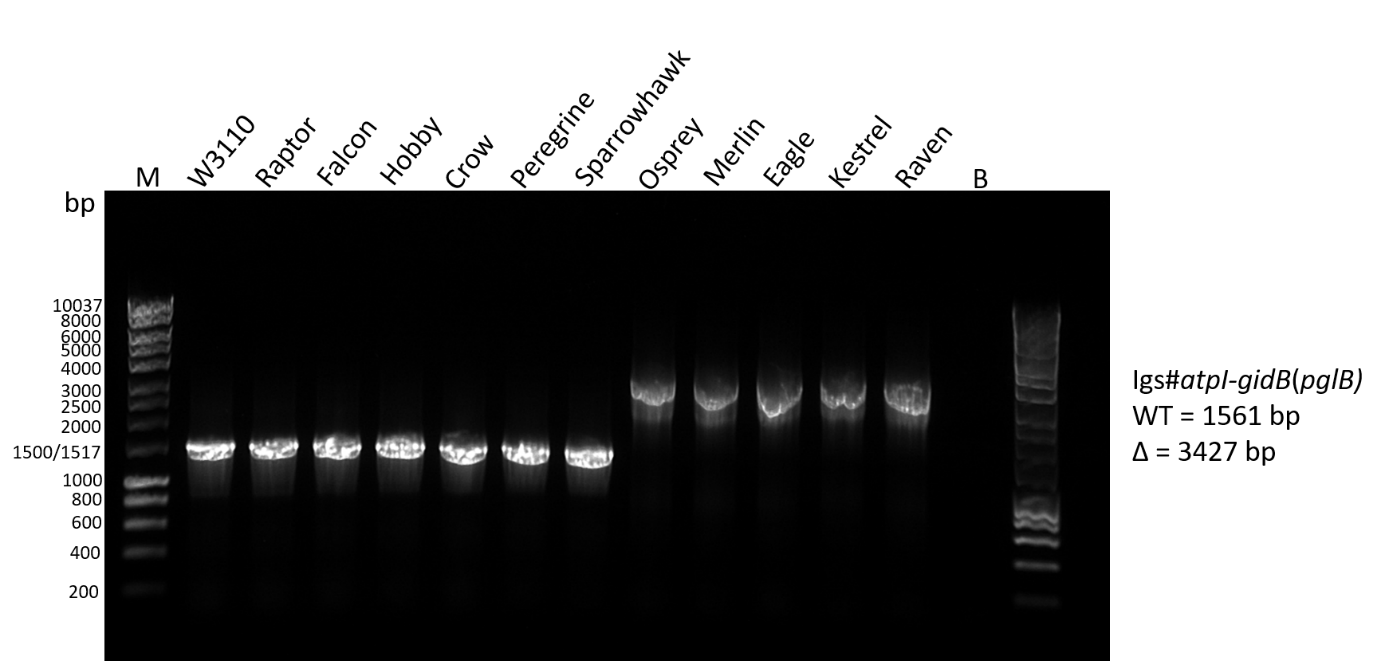


**Figure S1: PCR Verification of mutations within strains.**

Overnight cultures of each strain were lysed using Chelex 100 (BioRad). PCR was performed with Platinum Green Hot start PCR mastermix (Invitrogen), in 20 µl reaction, according to manufacturer’s instructions, using Tm determined by NEB Tm calculator. 10 µl was run on an agarose gel alongside Hyperladder 1 Kb marker (Bioline) and visualized with gelRed nucleic acid stain (biotium). M = Marker. B = negative control no template PCR.

**Growth curves**


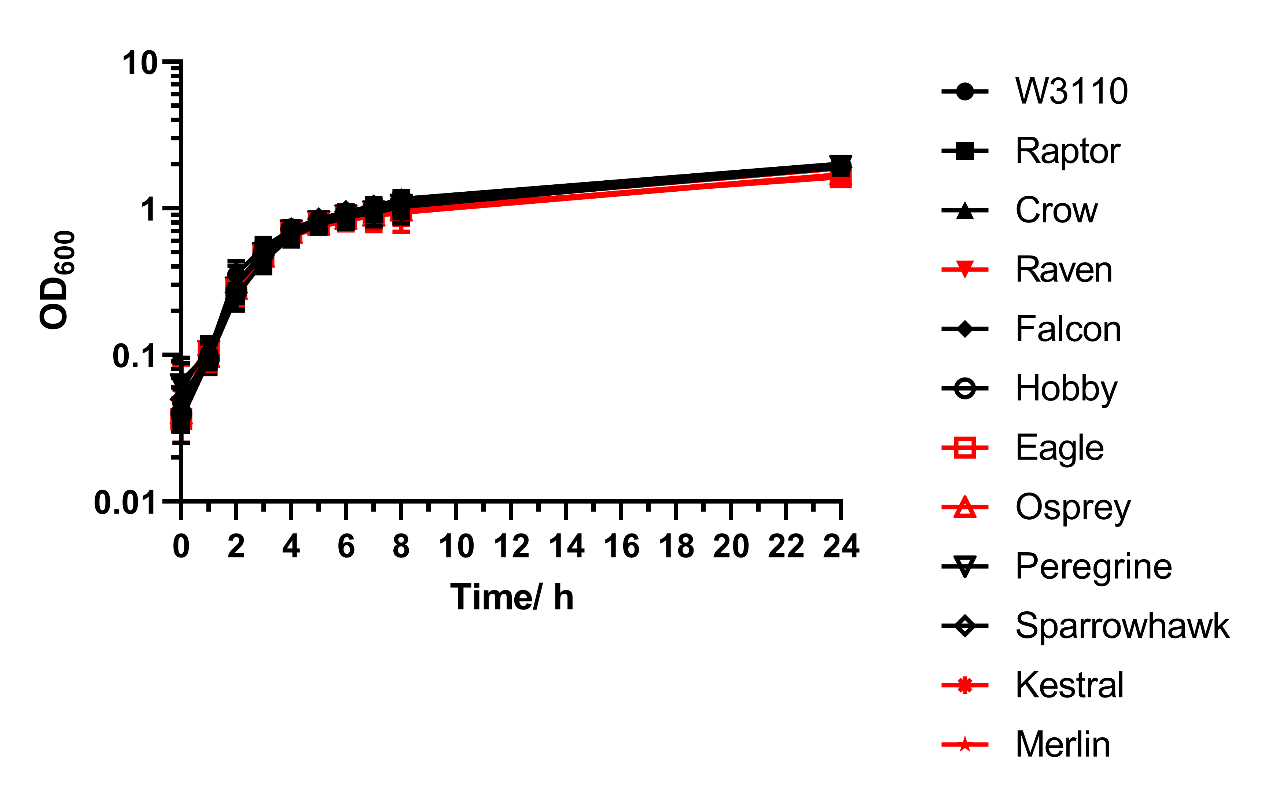


**Figure S2: Growth curve of all strains.**

Overnight cultures of all strains were used to inoculate fresh LB broth to an OD_600_ of 0.03. Cultures were incubated at 37 °C with shaking and OD_600_ measurements taken at regular intervals with a final reading taken at 24 h. Strains did not contain plasmids. Strains depicted in red contain *pglB* and grow slightly slower than those without and reach a slightly lower final OD_600_. W3110 is included as a wild type comparison. The experiment was performed in triplicate and the data shown are mean values with error bars depicting standard error of the mean.

**TLR4 activation assay using HEKBlue cells**


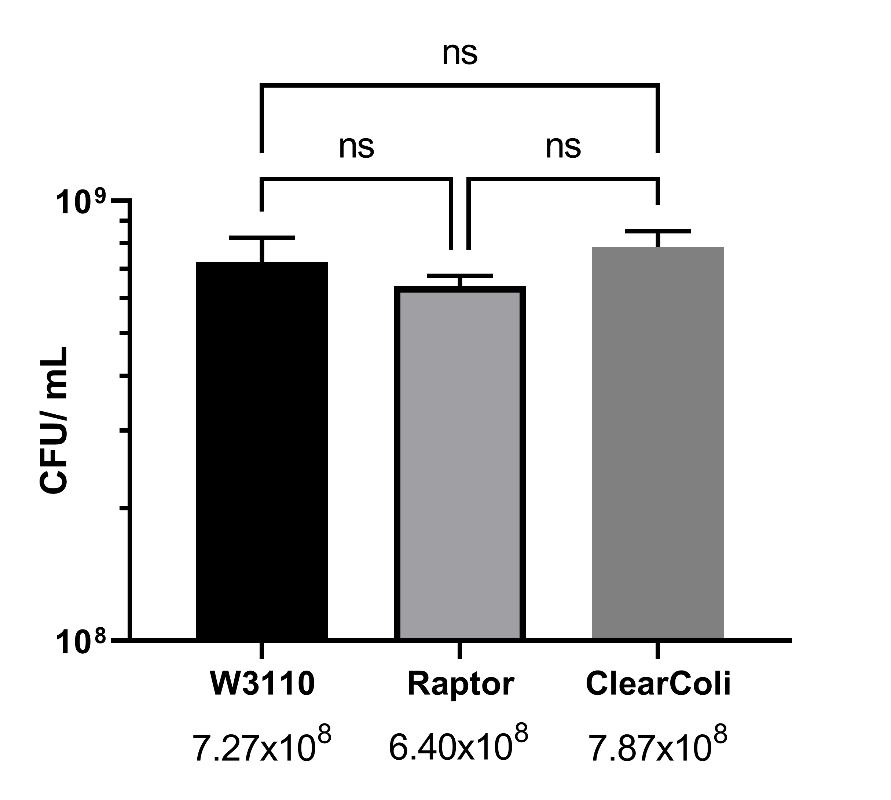


**Figure S3: Colony forming units for TLR4 activation assay.**

Overnight cultures of W3110, Raptor and ClearColi strains were matched for OD_600_ value, serially diluted in PBS, and plated on LB agar plates in triplicate. The plates were grown at 37°C overnight before counting and determining CFU/ ml. The average results were plotted, and a one-way ANOVA with Tukey’s multiple comparison test was used to determine whether the difference was significant. The OD matched samples were then further diluted in HEKblue cell culture media prior to the TLR4 activation assay.

**Repair of frame shift in wzE**


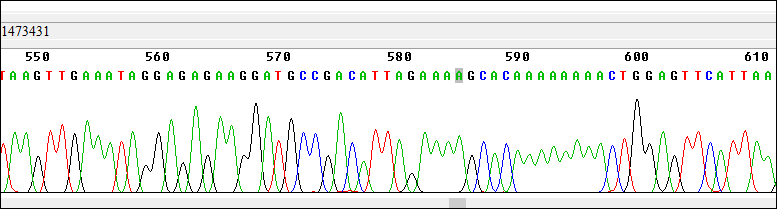


GTTGAAATAGGAGAGAAGGATGCCGACATTAGAAA-AGCACaaaaaaaaCTGGAGTTCAT

||||||||||||||||||||||||||||||||||| ||||||||||||||||||||||||

GTTGAAATAGGAGAGAAGGATGCCGACATTAGAAATAGCACAAAAAAAACTGGAGTTCAT

**Figure S4: Frame shift in wzE within the pTarget recombination construct Δ*wzzB*(*wzD-wzE).***

Chromatogram of sequence viewed in Chromas software (Technelysium Pty Ltd, Australia) with the base after deletion highlighted. Alignment of the pTarget recombination construct sequence (top) against the *S. pneumoniae* TIGR4 genome sequence (bottom) with ATG start codon of wzE in green. A thymine (T) is missing from the pTarget recombination construct sequence which causes a frame shift in the open reading frame of wzE.

**Conjugation test for new strains with chromosomally integrated *pglB*.**


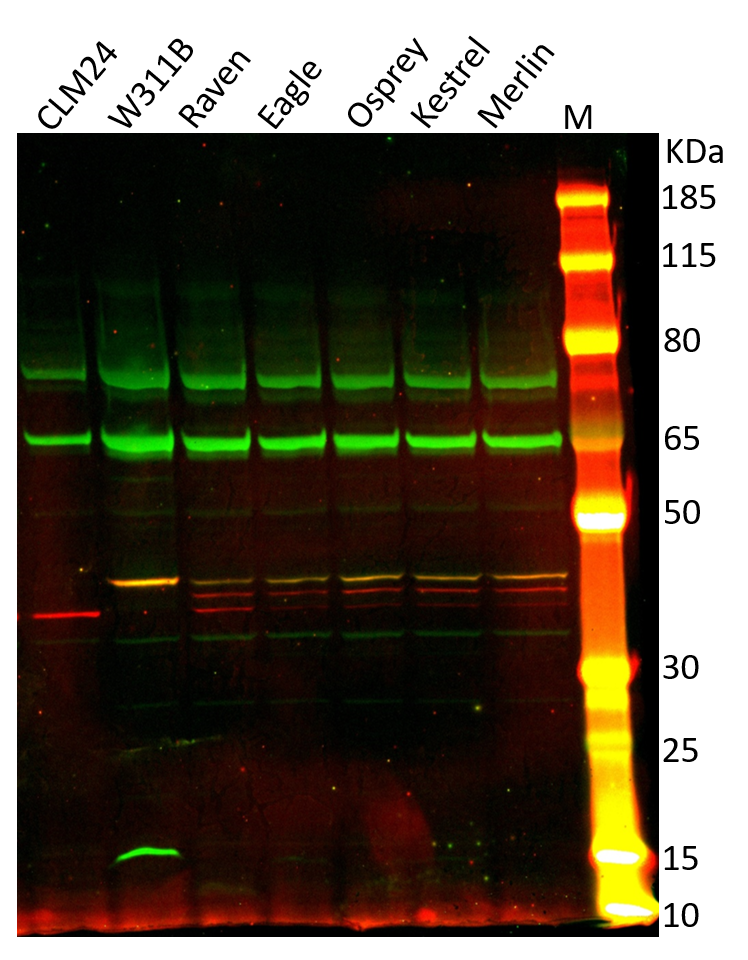


**Figure S5: Conjugation of *C. jejuni* heptasachcaride to AcrA.**

Overnight culture was used to seed 2YP broth to OD600 of 0.03. After 4 hrs 1 mM IPTG was added to W311B pPgl::pglB pWA2. After 24 hr growth at 28°C cells were harvested, matched to OD600 20 and lysed with FastPrep homogenizer. 15 µl lysate was loaded on a Bolt 4-12% bis-tris gel with MOPS buffer. After transfer to nitrocellulose membrane protein and glycan were detected using mouse anti-His monoclonal antibody (Abcam, UK) and HR6 antiserum (S. Amber and M. Aebi, unpublished data) respectively. Secondary goat anti-rabbit IgG IRDye 800 and goat anti-mouse IgG IRDye 680 conjugates were used tp generate fluorescent signal which was detected using an Odyssey LI-COR detection system (LI-COR Biosciences UK Ltd.). Green channel is for Glycan, Red for protein. All strains contained pACYCPgl::pglB and pWA2. CLM24 does not contain a functional PglB transferase so only unclycosylated AcrA is visible. W311B contains chromosomally integrated *pglB* under a P_tac_ promoter and the remaining strains contain integrated *pglB* under an Anderson X10 promoter. M = Marker (PageRuler plus prestained protein ladder – Fisher). AcrA runs at 40 KDa.

# References

Bachmann, B. J. (1996). “Derivations and genotypes of some mutant derivatives of Escherichia coli K-12." In Escherichia coli and Salmonella: cellular and molecular biology, 2nd ed. ASM Press, Washington, DC: 2460-2488.

Dykxhoorn, D. M., R. St Pierre and T. Linn (1996). A set of compatible tac promoter expression vectors. Gene **177**(1-2): 133-136.

Feldman, M. F., M. Wacker, M. Hernandez, P. G. Hitchen, C. L. Marolda, M. Kowarik, H. R. Morris, A. Dell, M. A. Valvano and M. Aebi (2005). Engineering N-linked protein glycosylation with diverse O antigen lipopolysaccharide structures in *Escherichia coli*. Proceedings of the National Academy of Sciences of the United States of America **102**(8): 3016-3021.

Herbert, J. A., E. J. Kay, S. E. Faustini, A. Richter, S. Abouelhadid, J. Cuccui, B. Wren and T. J. Mitchell (2018). Production and efficacy of a low-cost recombinant pneumococcal protein polysaccharide conjugate vaccine. Vaccine **36**(26): 3809-3819.

Jiang, Y., B. Chen, C. Duan, B. Sun, J. Yang and S. Yang (2015). Multigene editing in the *Escherichia coli* genome via the CRISPR-Cas9 system. Appl Environ Microbiol **81**(7): 2506-2514.

Kay, E. J., L. E. Yates, V. S. Terra, J. Cuccui and B. W. Wren (2016). Recombinant expression of *Streptococcus pneumoniae* capsular polysaccharides in *Escherichia coli*. Open Biol **6**(4): 150243.

Kovach, M. E., P. H. Elzer, D. S. Hill, G. T. Robertson, M. A. Farris, R. M. Roop, 2nd and K. M. Peterson (1995). Four new derivatives of the broad-host-range cloning vector pBBR1MCS, carrying different antibiotic-resistance cassettes. Gene **166**(1): 175-176.

Linton, D., N. Dorrell, P. G. Hitchen, S. Amber, A. V. Karlyshev, H. R. Morris, A. Dell, M. A. Valvano, M. Aebi and B. W. Wren (2005). Functional analysis of the *Campylobacter jejuni* N-linked protein glycosylation pathway. Mol Microbiol **55**(6): 1695-1703.

Samaras, J. J., M. Mauri, E. J. Kay, B. W. Wren and M. Micheletti (2021). Development of an automated platform for the optimal production of glycoconjugate vaccines expressed in *Escherichia coli*. Microbial Cell Factories **20**(1): 104.
